# Local genes for local bacteria: Evidence of allopatry in the genomes of transatlantic *Campylobacter* populations

**DOI:** 10.1111/mec.14176

**Published:** 2017-06-19

**Authors:** Ben Pascoe, Guillaume Méric, Koji Yahara, Helen Wimalarathna, Susan Murray, Matthew D. Hitchings, Emma L. Sproston, Catherine D. Carrillo, Eduardo N. Taboada, Kerry K. Cooper, Steven Huynh, Alison J. Cody, Keith A. Jolley, Martin C. J. Maiden, Noel D. McCarthy, Xavier Didelot, Craig T. Parker, Samuel K. Sheppard

**Affiliations:** ^1^ The Milner Centre for Evolution Department of Biology and Biochemistry Bath University Claverton Down Bath UK; ^2^ MRC CLIMB Consortium Bath UK; ^3^ Department of Bacteriology II National Institute of Infectious Diseases Tokyo Japan; ^4^ Swansea University Medical School Swansea University Swansea UK; ^5^ Department of Zoology University of Oxford Oxford UK; ^6^ Bureau of Microbial Hazards Health Canada Ottawa ON Canada; ^7^ Canadian Food Inspection Agency Ottawa ON Canada; ^8^ National Microbiology Laboratory at Lethbridge Public Health Agency of Canada Lethbridge AB Canada; ^9^ Department of Biology California State University Northridge Northridge CA USA; ^10^ Produce Safety and Microbiology Research Unit Agricultural Research Service US Department of Agriculture Albany CA USA; ^11^ NIHR Health Protection Research Unit in Gastrointestinal Infections Oxford UK; ^12^ University of Warwick Coventry UK; ^13^ Department of Infectious Disease Epidemiology Imperial College London London UK

**Keywords:** allopatry, *Campylobacter*, genomics, phylogeny, recombination, source attribution

## Abstract

The genetic structure of bacterial populations can be related to geographical locations of isolation. In some species, there is a strong correlation between geographical distance and genetic distance, which can be caused by different evolutionary mechanisms. Patterns of ancient admixture in *Helicobacter pylori* can be reconstructed in concordance with past human migration, whereas in *Mycobacterium tuberculosis* it is the lack of recombination that causes allopatric clusters. In *Campylobacter*, analyses of genomic data and molecular typing have been successful in determining the reservoir host species, but not geographical origin. We investigated biogeographical variation in highly recombining genes to determine the extent of clustering between genomes from geographically distinct *Campylobacter* populations. Whole‐genome sequences from 294 *Campylobacter* isolates from North America and the UK were analysed. Isolates from within the same country shared more recently recombined DNA than isolates from different countries. Using 15 UK/American closely matched pairs of isolates that shared ancestors, we identify regions that have frequently and recently recombined to test their correlation with geographical origin. The seven genes that demonstrated the greatest clustering by geography were used in an attribution model to infer geographical origin which was tested using a further 383 UK clinical isolates to detect signatures of recent foreign travel. Patient records indicated that in 46 cases, travel abroad had occurred <2 weeks prior to sampling, and genomic analysis identified that 34 (74%) of these isolates were of a non‐UK origin. Identification of biogeographical markers in *Campylobacter* genomes will contribute to improved source attribution of clinical *Campylobacter* infection and inform intervention strategies to reduce campylobacteriosis.

## 1 INTRODUCTION

Geographical structure is well documented in bacteria and analysing genetic variation among isolates can provide information about the global spread of important pathogens. For example, after spreading with Neolithic human hosts (Comas et al., 2013), lineages of *Mycobacterium tuberculosis* populations can be classified into geographical groups based upon local genetic diversification of DNA sequences (Achtman, 2008; Gagneux & Small, 2007). Phylogeographical structure has also been observed in the human gastric bacterium *Helicobacter pylori,* where a rapidly evolving genome with high levels of horizontal gene transfer (HGT) allows the reconstruction of recent human migrations to the extent that genetic admixture among the bacteria reflects interactions among human populations (Falush et al., 2003; Moodley et al., 2009).

*Mycobacterium tuberculosis* and *H. pylori* are primarily human pathogens. However, in the foodborne pathogen *Campylobacter,* animals are the principal reservoir for human infection. International trade, particularly in agricultural animals including chicken and poultry products, provides a vehicle for global spread. In this case, local phylogeographical signals can be weakened not only by the rapid movement of lineages around the world, but also by genomic changes that occur within the reservoir host. This may make it difficult to attribute the country of origin based on the *Campylobacter* isolate genome alone. Sequence‐based analyses have shown that populations of the main human disease‐causing *Campylobacter* species, *C. jejuni* and *C. coli,* are highly structured into clusters of related lineages, which can be identified by MLST as clonal complexes (CCs). Members of CCs share four or more MLST alleles with a predefined central genotype, which gives the CC its name; for example, ST‐21 defines CC‐21 (Dingle, Colles, Falush, & Maiden, 2005; Sheppard, Dallas et al., 2010). In *C. jejuni,* host‐associated clonal complexes can be identified based upon the frequency with which particular genotypes are isolated from different hosts (Sheppard et al., 2011, 2014). Many of these lineages are globally distributed (Sheppard, Colles et al., 2010) but despite this strong host signal, there is evidence for phylogeographical structuring and the proliferation of distinct lineages in different countries (Asakura et al., 2012; McTavish et al., 2008).

Horizontal gene transfer in recombining bacteria, such as *Campylobacter* (Sheppard, McCarthy, Falush, & Maiden, 2008; Sheppard, Didelot, Jolley et al., 2013; Wilson et al., 2008), can provide information about ecological differences between lineages. For example, when a *Campylobacter* lineage transfers to a new animal host it may acquire DNA from the resident population by HGT. This has been shown in host generalist *C. jejuni* lineages isolated from chicken that sometimes contain alleles that originated in chicken specialist genotypes (McCarthy et al., 2007; Wilson et al., 2008). In this study, we applied comparable approaches to investigate whether HGT can lead to signatures of recombination that discriminate between isolates from North America and the UK using genomic data. Using matched pairs of North American and UK isolates, we identify genes that are prone to recombination, and will therefore pick up local DNA more rapidly, and hypothesize that these genes may acquire a biogeographical signal.

## 2 MATERIALS AND METHODS

### 2.1 Bacterial isolates and genome sequencing

A total of 294 sequenced isolates were analysed, of which 131 genomes were generated in this study and augmented by 163 previously published genomes (Sheppard, Didelot, Jolley et al., 2013; Sheppard, Didelot, Meric et al., 2013; Sheppard et al., 2014). Sequencing reads for all genomes sequenced in this study are available from the NCBI short read archive associated with BioProject: PRJNA312235. All assembled genomes used in this study can also be downloaded from FigShare (https://doi.org/10.6084/m9.figshare.4906634).

#### 2.1.1 Canadian isolates

Isolates were collected from chicken and bovine faecal samples between July 2004 and July 2006 from farms at diverse locations in Alberta. Samples were placed on ice and processed within 6 h as previously described (Jokinen et al., 2010). Approximately 5 g of faecal matter was mixed with 5 ml of phosphate buffered saline (PBS) to form uniform slurry. One‐millilitre aliquots of the PBS‐faecal samples were added to 20 ml of Bolton broth containing 5% (v/v) lysed horse blood and selective supplement (Diergaardt, Venter, Spreeth, Theron, & Brozel, 2004) and incubated at 42°C for 24 hr under microaerobic conditions prior to plating 20 μl onto supplemented charcoal cefoperazone deoxycholate agar (CCDA). The plates were incubated for a further 48 hr at 42°C. Human samples were acquired from clinical laboratories in three Canadian provinces. These were replated from frozen glycerol stocks and the DNA extracted as described below.

Presumptive *Campylobacter* colonies were cultured onto blood agar plates and tested using biochemical oxidase and catalase tests. *Campylobacter* species identification was performed using a multiplex PCR assay that included 16S rRNA gene primers and *C. jejuni* (*mapA*) and *C. coli*‐specific (*ceuE*) primers (Denis et al., 1999). Positive *Campylobacter* isolates were subcultured on Mueller–Hinton agar and genomic DNA was extracted using the Wizard Genomic DNA Purification Kit as per the manufacturer's instructions (Promega, Madison, WI, USA). DNA integrity was checked on an agarose gel and purity and concentration determined by optical density. Purified genomic DNA was sent to Canada's Michael Smith Genome Sciences Centre (Vancouver, Canada) and sequenced using the Illumina HiSeq 2000 platform. Sequence reads were assembled into contigs using the spades assembler (v3.0) (Bankevich et al., 2012).

#### 2.1.2 US isolates

Isolates were collected from cattle faecal samples between December 2008 and June 2010 from diverse locations within the Salinas Valley watershed, California. Samples were placed on ice and processed within 12 hr. Cattle faeces were inoculated into a six‐well microtitre plate containing 6 ml 1× Anaerobe Basal Broth (Oxoid) amended with Preston supplement (when reconstituted consists of amphotericin B (10 μg/ml), rifampicin (10 μg/ml), trimethoprim lactate (10 μg/ml) and polymyxin B (5 UI/ml) (Oxoid)), using a sterile cotton swab. Microtiter plates were placed inside plastic ZipLoc bags filled with a microaerobic gas mixture (1.5% O_2_, 10% H_2_, 10% CO_2_ and 78.5% N_2_) and incubated for 24 hr at 37°C, while shaking at 40 rpm. Subsequently, 10 μl of these enrichment cultures was plated onto anaerobe basal agar (ABA, Oxoid) plates, amended with 5% laked horse blood and CAT supplement (cefoperazone (8 μg/ml), amphotericin B (10 μg/ml) and teicoplanin (4 μg/ml) (Oxoid)). All plates were then incubated under microaerobic conditions at 37°C for 24 hr. Bacterial cultures were passed through 0.2‐μm mixed cellulose ester filters onto ABA plates and incubated at 37°C under microaerobic conditions. After 24 hr, single colonies were streaked onto fresh ABA plates and incubated 24–48 hr at 37°C for purification.

DNA was extracted from a pure culture colony using the Wizard Genomic DNA Purification Kit (Promega). *Campylobacter* species was identified by 16S rDNA sequencing, using the primer pairs as described by Lane (1991). Genome sequencing was performed on an Illumina MiSeq sequencer using the KAPA Low‐Throughput Library Preparation Kit with Standard PCR Amplification Module (Kapa Biosystems, Wilmington, MA, USA), following the manufacturer's instructions except for the following changes; 750 ng DNA was sheared at 30 psi for 40 s and size selected to 700–770 bp following Illumina protocols. Standard desalted TruSeq LT and PCR primers were ordered from Integrated DNA Technologies (Coralville, IA) and used at 0.375 and 0.5 μm final concentrations, respectively. PCR was reduced to 3–5 cycles. Libraries were quantified using the KAPA Library Quantification Kit (Kapa), except with 10 μl volume and 90‐s annealing/extension PCR, and then pooled and normalized to 4 nM. Pooled libraries were requantified by ddPCR on a QX200 system (Bio‐Rad, Hercules, CA, USA), using the Illumina TruSeq ddPCR Library Quantification Kit and following the manufacturer's protocols, except with an extended 2‐min annealing/extension time. Libraries were sequenced using a 2 × 250 bp paired end v2 reagent kit on a MiSeq instrument (Illumina, San Diego, CA, USA) at 13.5 pm, following the manufacturer's protocols. Genomes were assembled using the Roche Newbler assembler (version 2.3).

#### 2.1.3 Published isolates

We augmented the collection of isolates sequenced in this study with 163 previously published *Campylobacter* isolate genomes from Canada, the United States and the UK collected between 1980 and 2012 from a range of sources, including cattle (54), chicken (80), pig (9), environmental (49), wild bird species (12) and human clinical cases (73) (Table S1; Sheppard, Didelot, Jolley et al., 2013; Sheppard, Didelot, Meric et al., 2013; Sheppard et al., 2014).

#### 2.1.4 UK clinical test isolates

In addition to this collection of sequenced and publicly available *Campylobacter* genomes, we used a further 383 clinical samples collected from the John Radcliffe Hospital in Oxford between June and October 2011 as a test data set to attribute source according to geography (Table S2; Cody et al., 2013). These genomes were downloaded from http://pubmlst.org/campylobacter/.

### 2.2 Population structure

Isolate genomes were archived on an open‐source bigsdb database which identifies gene presence and allelic variation by comparison with a reference locus list (Jolley & Maiden, 2010; Meric et al., 2014; Sheppard, Jolley, & Maiden, 2012). This list comprises 1,623 locus designations from the annotated genome of *C. jejuni* strain NCTC11168 (GenBank accession no. NC_002163.1) (Gundogdu et al., 2007; Parkhill et al., 2000). Reference loci were identified in each of the 294 isolate genomes using blast. Loci were recorded as present if the sequence had ≥70% nucleotide identity over ≥50% of the gene length. Each gene was aligned individually using mafft (Katoh, Misawa, Kuma, & Miyata, 2002) and concatenated into a single multi‐FASTA alignment file for each isolate for a total alignment of 1,585,605 bp. Phylogenetic trees were constructed from a whole‐genome alignment of *C. jejuni* (*n* = 229) and *C. coli* (*n* = 55) isolates based on 103,878 and 806,657 variable sites, respectively, using fasttree (version 2) and an approximation of the maximum‐likelihood algorithm (Kumar, Stecher, & Tamura, 2016; Tamura, Stecher, Peterson, Filipski, & Kumar, 2013).

### 2.3 Selection of isolate pairs

To minimize the effect of host adaptation and maximize the opportunity of identifying genetic signatures of geographical separation, a subset of 15 isolate pairs were chosen based upon their phylogenetic clustering. In each case, isolate pairs contained one Canadian and one UK isolate of the same clonal complex sampled from the same host species. Paired isolates shared 1,378 genes resulting in a core‐genome alignment of 1,287,560 bp.

### 2.4 Analysis of co‐ancestry and inference of recombination hot regions

The co‐ancestry of the paired isolates was inferred based on whole‐genome sequences using chromosome painting and finestructure (Lawson, Hellenthal, Myers, & Falush, 2012), as previously described (Yahara et al., 2013). chromopainter (version 0.02) was used to infer the number of DNA “chunks” donated from a donor to a recipient for each recipient haplotype, and the results summarized in a co‐ancestry matrix indicating average isolate similarity across the entire genome. finestructure was then used for 100,000 iterations of both the burn‐in and Markov chain Monte Carlo (MCMC) chain to cluster individuals based on the co‐ancestry matrix. The results are visualized as a heat map with each cell indicating the proportion of DNA “chunks” a recipient receives from each donor.

The time to the most recent common ancestor (TMRCA) of each pair was estimated using the model described in Didelot et al. (2013) and summarized here briefly. Pairs of genomes share a common ancestor ***t*** years ago and have been subject to mutation at a rate **μ** and recombination at rate **ρ**. The mutation rate of 2.9 × 10^−5^ per site per year was used as reported in (Sheppard, Dallas et al., 2010), which is similar to the rates estimated in Wilson et al. (2008, 2009). The effect of recombination is to introduce a high density of polymorphism similar to the ClonalFrame model (Didelot & Falush, 2007; Didelot & Wilson, 2015) but with the advantage that this density can vary between recombination events to reflect differences in evolutionary distance between donors and recipients (Didelot et al., 2013; Morelli et al., 2010). In each pairwise comparison, the TMRCA and recombination rate parameters are estimated based on a core‐genome alignment, with 95% credibility intervals.

### 2.5 Epidemiological markers of geographical clustering

Neighbour‐joining phylogenetic trees were constructed for all genes that demonstrated an average of above 1% pairwise nucleotide diversity across all 15 pairs of isolates. Individual gene phylogenies were constructed in mega for all 57 genes. Isolates were assigned to a putative source population based on the seven highly recombining genes that showed the greatest level of clustering by geography. Probabilistic assignment of geographical source is based on the allele frequencies in the reference population data sets for each of the seven loci. This analysis was performed using structure, a Bayesian model‐based clustering method designed to infer population structure and assign individuals to populations using multilocus genotype data (Pritchard, Stephens, & Donnelly, 2000; Sheppard, Colles et al., 2010). Canadian and US isolates were combined as a North American population for comparison with UK isolates.

### 2.6 Attribution of clinical isolates to country based on seven geographically segregating genes

The source attribution model was tested with isolates of a known source. Self‐assignment of a random subset of the comparison data set was conducted by removing a third of the isolates from each candidate population (*n* = 73). The remainder were used as the reference set (78 North American isolates to compare with 68 UK isolates). Structure was run for 100,000 iterations following a burn‐in period of 10,000 iterations using the no admixture model to assign individuals to putative populations. The assignment probability for each source was calculated for each isolate individually and were attributed to origin populations when the attribution probability was >0.50.

## 3 RESULTS

**Figure 1:**
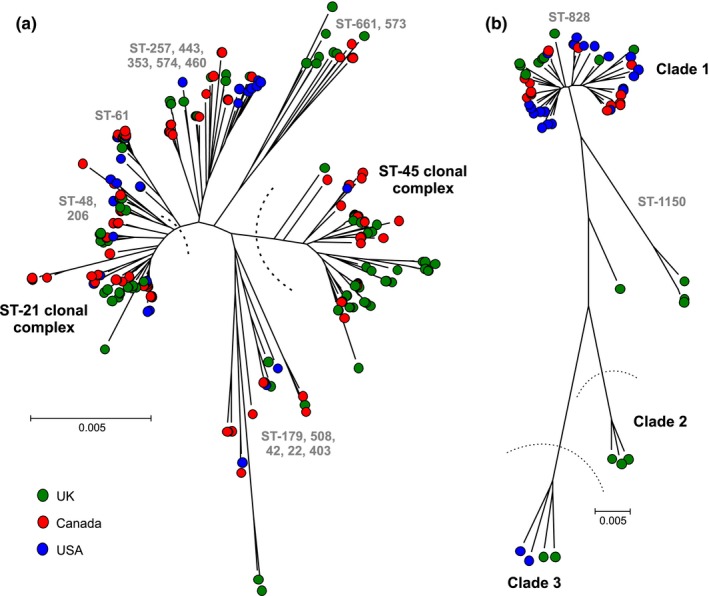
Population structure of *Campylobacter* isolates used in this study. Phylogenetic trees were constructed from a whole‐genome alignment of (a) *C. jejuni* (*n* = 229) and (b) *C. coli* (*n* = 55) isolates based on 103,878 and 806,657 variable sites, respectively, using an approximation of the maximum‐likelihood algorithm (Kumar et al., 2016; Tamura et al., 2013). Leaves on the tree are coloured by source country, the UK (green circles), Canada (red) and the United States (blue). Ancestral *C. coli* clades (1, 2 and 3) (Sheppard, Dallas et al., 2010) are annotated and common clonal complexes (CC) based on four or more shared alleles in seven MLST housekeeping genes (Dingle et al., 2005)

Core genomes of isolates from North America and the UK were compared, and there was no observable clustering by country or continent on a neighbour‐joining tree (Figure 1). STs sampled in both *Campylobacter* populations belonged to clonal complexes that can be classified as specialist and host generalist based upon the frequency at which they have been isolated from different hosts. These included chicken specialist clonal complexes CC‐257, CC‐283, CC‐353, CC‐354, CC‐443, CC‐573, CC‐574 and CC‐661, cattle specialist CC‐61 and CC‐42, and host generalist CC‐21, CC‐45, CC‐206 and CC‐48 (Figure 1 and Table S1).

### 3.1 Matched isolates share more common ancestry with isolates from the same country

**Figure 2:**
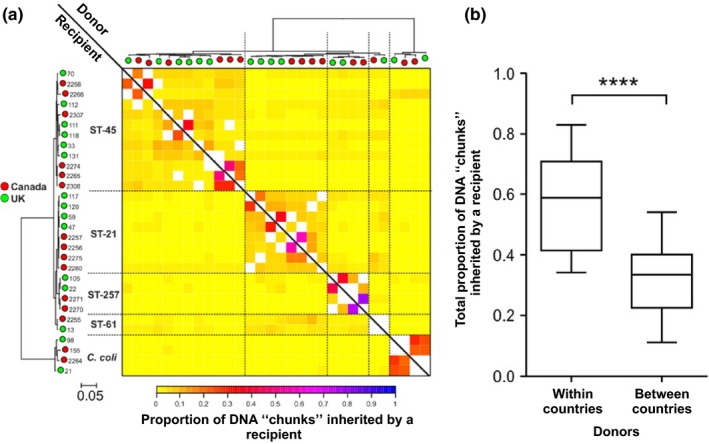
Co‐ancestry matrix with population structure and genetic flux. (a) The colour of each cell of the matrix indicates proportion of DNA chunks in a recipient genome (row) from a donor genome (column). The colour ranges from little (yellow) to a large amount of DNA from the donor strain (blue). Diagonal white cells indicate chunks of DNA that are shared between the pairs of isolates and masked in the comparison in (b). The trees above and to the left show clustering of the paired isolates with leaves coloured by source country (the UK in green, Canada in red). (b) Boxplot comparing total proportion of chunks of DNA inherited by a recipient from donors either within or between countries. The total proportion is significantly higher for chunks of DNA from donor strains of the same country compared to those from different countries (*p *< 10^−9^, Wilcoxon rank‐sum test)

To minimize the effect of host adaptation and maximize the opportunity of identifying genetic signatures of geographical separation, a subset of 15 isolate pairs were chosen based upon their phylogenetic clustering with less than 1,200 bp difference in 1,378 core‐genome loci. In each case, isolate pairs contained one Canadian and one UK isolate of the same clonal complex sampled from the same host species (Table 1). The co‐ancestry of the paired isolates was inferred based on core‐genome alignments using chromosome painting and finestructure (Lawson et al., 2012) (Yahara et al., 2013) (Figure 2). The total proportion of DNA “chunks” in a recipient from isolates within the same country (median 0.59) was significantly higher than that from isolates from different countries (median 0.33; *p *< 10^−9^, Wilcoxon rank‐sum test).

**Table 1:** Isolate pairs matched by clonal complex and host

Pair	Isolate	Origin	Host	MLST genes							Clonal complex
				aspA	glnA	gltA	glyA	pgm	tkt	uncA	
1	2,256	Canada	Cattle	2	1	1	3	2	1	5	ST‐21
	47	The UK	Cattle	2	1	1	3	2	1	5	ST‐21
2	2,280	Canada	Human	2	1	1	3	2	1	5	ST‐21
	117	The UK	Human	2	1	1	3	2	1	5	ST‐21
3	2,271	Canada	Chicken	9	2	4	62	4	5	17	ST‐257
	22	The UK	Chicken	9	2	4	62	4	5	6	ST‐257
4	2,274	Canada	Duck	4	7	10	4	1	7	1	ST‐45
	131	The UK	Duck	4	7	10	4	1	7	1	ST‐45
5	2,258	Canada	Chicken	4	7	10	4	1	7	1	ST‐45
	112	The UK	Chicken	4	7	10	4	1	7	1	ST‐45
6	2,306	Canada	Human	4	7	10	4	1	7	1	ST‐45
	33	The UK	Human	4	7	10	4	1	7	1	ST‐45
7	2,255	Canada	Cattle	1	4	2	2	6	3	17	ST‐61
	13	The UK	Cattle	1	4	2	2	6	3	17	ST‐61
8	2,264	Canada	Chicken	33	39	30	203	113	47	17	ST‐828
	21	The UK	Chicken	33	39	30	82	104	43	17	ST‐828
9	2,257	Canada	Cattle	2	1	1	3	2	1	5	ST‐21
	59	The UK	Cattle	2	1	1	3	2	1	5	ST‐21
10	2,275	Canada	Human	2	1	1	3	2	1	5	ST‐21
	120	The UK	Human	2	1	1	3	2	1	5	ST‐21
11	2,270	Canada	Chicken	9	2	4	62	4	5	17	ST‐257
	105	The UK	Chicken	9	2	4	62	4	5	6	ST‐257
12	2,265	Canada	Chicken	4	7	10	4	1	7	1	ST‐45
	111	The UK	Chicken	4	7	10	4	1	7	1	ST‐45
13	2,266	Canada	Chicken	4	7	10	4	1	7	1	ST‐45
	70	The UK	Chicken	4	7	10	4	1	7	1	ST‐45
14	2,307	Canada	Human	4	7	10	4	1	7	1	ST‐45
	118	The UK	Human	4	7	10	4	1	7	1	ST‐45
15	155	Canada	Cattle	33	39	30	82	104	85	68	ST‐828
	98	The UK	Cattle	33	39	30	82	104	56	17	ST‐828

### 3.2 Matched isolates share recent common ancestors but have since experienced significant recombination

**Figure 3:**
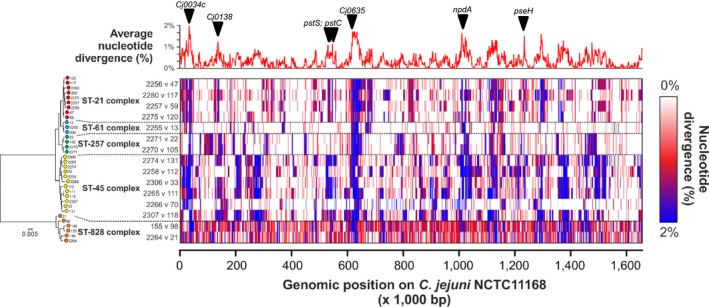
Pairwise comparison of nucleotide diversity in the core genome. Above: Estimated values of the per‐nucleotide statistic reflecting relative intensity of recombination at each site plotted along the NCTC11168 reference genome. Left: Core‐genome phylogeny of selected paired isolates (matched by CC and source host), with clonal complex indicated. Centre: Matrix of gene‐by‐gene pairwise comparison along the NCTC11168 reference genome of our selected pairs. Each row represents a pairwise comparison of selected paired of isolates. Each column is a gene from the NCTC11168 reference genome. Panels of the matrix are coloured based on nucleotide divergence for that gene in each pair: from no nucleotide diversity (0%, white), through some nucleotide diversity (~1%, red) to high levels of nucleotide diversity (up to 2%, blue). The per‐nucleotide scan of relative intensity of recombination is aligned with our gene‐by‐gene pairwise comparison of nucleotide diversity, and the location of seven putative epidemiological markers for geographical segregation is indicated

The estimated time since the most recent common ancestor (TMRCA) was calculated for each UK/American pair of genomes as previously described (Didelot et al., 2013), using the mutation rate of 2.9 × 10^−5^ per site per year reported in Sheppard, Dallas et al. (2010), which is consistent with estimates in Wilson et al. (2009). In each pairwise comparison, the level of divergence along the genome (Figure 3) was used to estimate the TMRCA and recombination rate, with 95% credibility intervals around these parameters (Table 2). All pairs were estimated to have shared ancestors between one and 5 years ago, with two exceptions, namely the two *C. coli* pairs, for which the TMRCA was around 25 years ago. The ratio r/m of rates at which recombination and mutation introduce polymorphism was estimated to be around 20–30 except in the two *C. coli* pairs with larger TMRCA, for which a smaller value was estimated around r/m = 4. Most existing r/m estimates have been calculated using seven MLST housekeeping genes (Vos & Didelot, 2009; Wilson et al., 2008, 2009). Other estimates have been derived through comparison of relatively small numbers of *Campylobacter* genomes (Llarena et al., 2016). Estimates of r/m can vary considerably depending on the isolate collection and the genes used in the analysis, for example, ranging from 0 to 100 among *Helicobacter* isolates within a human population from within a single settlement in South Africa (Didelot et al., 2013). Given the potential for sample‐dependent variation, the r/m estimates in this study are consistent with previous estimates. Further, variation in TMRCA estimates and r/m between *C. coli* compared to *C. jejuni* pairs in this study may reflect differences between the species, but more sampling of the *C. coli* population is necessary to investigate this further.

**Table 2:** Shared ancestry analysis and estimation of pairwise recombination rates. The time to the most recent common ancestor (TMRCA) for each selected pair was estimated with 95% confidence intervals (TMRCA‐CI). The ratio of rates at which recombination and mutation introduce polymorphism (r/m) was also calculated with 95% confidence intervals (r/m‐CI). In addition, the number of recombined genes (probability >95%) is also shown. The two *C. coli* pairs are shown in bold

Isolate pair	TMRCA	TMRCA‐CI	r/m	r/m‐CI	Definitely recombined genes (*p* > .95)
2,256 vs. 47	2.8	[2.5;3.2]	23.1	[20.2;26.3]	210
2,280 vs. 117	3.9	[3.2;4.5]	23.1	[19.0;28.3]	273
2,271 vs. 22	1.9	[1.6;2.3]	34.5	[28.8;39.6]	194
2,274 vs. 131	3.3	[2.9;3.9]	38.8	[32.0;43.6]	385
2,258 vs. 112	3.4	[3.0;3.8]	32.1	[28.4;37.0]	336
2,306 vs. 33	3.7	[3.2;4.2]	24.5	[21.0;27.9]	280
2,255 vs. 13	1.2	[1.0;1.5]	25.2	[20.1;30.2]	99
**2,264 vs. 21**	**22.7**	**[20.7;24.8]**	**3.9**	**[3.2;4.9]**	**187**
2,257 vs. 59	3	[2.5;3.5]	23.5	[19.3;27.8]	219
2,275 vs. 120	2.7	[2.3;3.1]	24.1	[20.4;27.9]	194
2,270 vs. 105	2.2	[1.9;2.5]	30.5	[26.6;34.8]	224
2,265 vs. 111	3.7	[3.3;4.2]	32.8	[28.6;36.7]	372
2,266 vs. 70	1.3	[1.1;1.5]	38	[33.4;41.4]	147
2,307 vs. 118	3.9	[3.4;4.6]	31.9	[26.2;37.4]	379
**155 vs. 98**	**27.1**	**[25.0;29.3]**	**3.6**	**[3.0;4.3]**	**236**

### 3.3 Highly recombining genes as markers of geographical attribution

A pairwise comparison of the matched pairs was used to quantify the level of divergence in each gene within the core genome (1,147 genes) of the paired isolates. Most genes showed low diversity, indicative of closely related pairs. Polymorphisms in genes with <1% divergence between pairs (white and red in Figure 3) are likely to be the result of mutation or recombination with a tract of DNA with high nucleotide identity, so that only one or two substitutions are visible. Genes with greater than 1% divergence between pairs are likely to have recombined as numerous substitutions have been introduced (blue in Figure 3). Fifty‐seven genes (e.g., *Cj0034c* and *Cj0635*) had a high level (>1%) of nucleotide divergence and high probability of recombination in all 15 pairs. This result did not arise just by chance: overall recombination was inferred in around 25% of the genes in each pair and so if recombination was random, the probability that all 15 pairs had recombined for a given gene would be extremely small (0.25^15^ = 9.3 × 10^−10^).

Individual gene trees were generated for these 57 genes from which the most recombination could be identified (Fig. S1). The seven genes that gave the clearest geographical clustering were used for further analysis of geographical attribution using Structure as previously described (Pritchard et al., 2000; Sheppard, Colles et al., 2010). A self‐test was performed on a subset of our isolate collection and in 76.7% of cases the source continent was correctly attributed. The percentages of correctly attributed isolates by population were not significantly different, at 76.9% for North America and 76.5% for the UK. Where an isolate was incorrectly attributed to a population there was a higher average reported attribution probability (0.85) in the case of UK isolates compared with North American isolates (0.67). When applied to the remainder of our isolate collection, the proportion of UK isolates correctly attributed to the UK reference population was 70%, while the proportion of North American isolates correctly attributed was slightly higher at 76%. This was not improved when using data from all 57 highly recombining genes as input for the attribution model in Structure (43% of UK and 72% of North American isolates correctly attributed).

### 3.4 Attribution of clinical isolates to country based on seven selected genes

**Figure 4:**
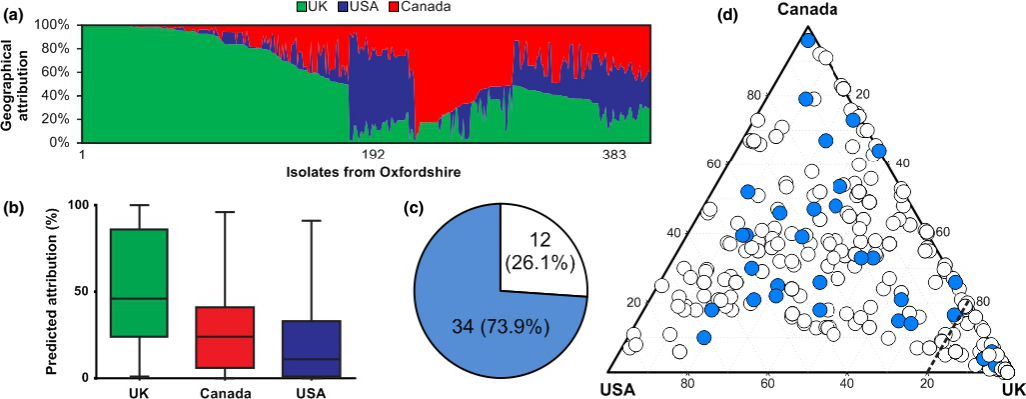
Assignment of human clinical cases of campylobacteriosis to origin country, including patients with history of recent foreign travel. (a) Assignment of human clinical cases of campylobacteriosis to origin country using epidemiological markers of biogeography and the Bayesian clustering algorithm Structure. Each isolate is represented by a vertical bar, showing the estimated probability that it comes from each of the putative source countries, including the UK (green), the United States (blue) and Canada (red). Isolates are ordered by attributed source. (b) Boxplots of predicted attribution probabilities for the three locations. (c) Isolates from Oxford clinical data set with declared history of recent foreign travel. The model correctly assigned 34 of 46 (73.9%) isolates to a non‐UK origin. (d) Attribution of Oxford clinical isolates between UK, US and Canadian source populations. Isolates with declared recent foreign travel are shown in blue

The same geographical attribution model was applied to 383 clinical *C. jejuni* isolates from the Oxfordshire *Campylobacter* Surveillance Study in the UK, accessed via pubMLST.org/campylobacter, and for which details of recent foreign travel were provided (Cody et al., 2013). The model correctly assigned 34 of the 46 (73.9%) isolates where recent foreign travel had previously been declared, to a non‐UK source of origin (Figure 4). In total, approximately half (47%) of the collected clinical isolates could be attributed to the UK.

## 4 DISCUSSION

Isolation of bacteria in different host species and barriers to recombination between populations overtime, can lead to population differentiation reflected in the genome. In *C. jejuni*, this can been at different levels, including the proliferation of certain lineages specific to a particular host species (Griekspoor et al., 2013; Sheppard, Colles et al., 2010 Sheppard et al., 2011). Increased frequency of host‐associated nucleotide substitutions in multiple lineages (that reflect adaptation to the host and drift) accumulate in physically isolated populations (Sheppard, Didelot, Meric et al., 2013). This host‐associated genetic structure can be informative for understanding the evolution of *C. jejuni* (Dearlove et al., 2016), but can also be used in a more practical way to identify the source of isolates causing human infection by identifying genomic signatures (resulting from adaptation or drift) in the infecting isolate that are associated with populations in particular reservoir hosts (Sheppard et al., 2009; Wilson et al., 2008). Quantitative source attribution models, based upon the probability that a particular clinical isolate originated in different reservoirs, have been widely used to estimate the risk of human infection from different food production animals and other sources (Colles et al., 2008; French et al., 2005; Griekspoor et al., 2013; Mullner et al., 2009; Roux et al., 2013; Sheppard et al., 2009; Thepault et al., 2017; Viswanathan et al., 2017) and have informed intervention strategies and public health policy (Cody et al., 2012, 2013).

The accuracy of probabilistic source attribution models is influenced by the degree to which indicative markers in the isolate genome, such as MLST locus alleles, can be placed within a source population. This is relatively straightforward for markers that segregate absolutely by source, but in *C. jejuni* and *C. coli* it is common that alleles are present in more than one population, but at different frequencies. In simple attribution models using MLST data, *C. jejuni* and *C. coli* isolates from chickens in the Netherlands, Senegal and the United States have been more closely related to UK chicken isolate populations rather than to populations from other host species in the same country (Sheppard, Colles et al., 2010). While genomic signatures of host association can transcend geographical structuring within *C. jejuni* and *C. coli* populations, there can be differences in the genotypes that are isolated from different countries (Asakura et al., 2012; Islam et al., 2014; Kivisto et al., 2014; Mohan et al., 2013; Prachantasena et al., 2016). This presents challenges, not only for attributing the source of infections among travellers returning from foreign locations (Mughini‐Gras et al., 2014), but also for understanding disease epidemiology in the context of a global food industry.

Following the occupation of a new niche *C. jejuni* and *C. coli* can acquire DNA signatures through recombination (Sheppard et al., 2008; Sheppard, Didelot, Jolley et al., 2013; Wilson et al., 2009) and local DNA signatures via HGT, from resident strains. To quantify the extent to which isolates from the same country share DNA sequence, we compared 15 isolate pairs from different countries that, to minimize the effect of clonal inheritance and host‐associated variation, were matched by both clonal complex and source. The predicted ancestry of co‐inherited SNPs was nearly twice as high among isolates from same country compared to those from different countries. While this represents a relatively weak signal of geographical association, compared to host association, there was a quantifiable local (national) signal that can be used to investigate geographical clustering.

As recombination introduces more nucleotide substitutions than during mutation in *C. jejuni* and *C. coli* (Morelli et al., 2010; Webb & Blaser, 2002; Wilson et al., 2009), genes with evidence of elevated recombination rates, which share a gene pool, will more rapidly acquire local signals of sequence variation than genes with lower recombination rates. These genes represent potential targets for use as biogeographical epidemiological markers. Pairwise isolate comparison revealed that nucleotide divergence was <1% across the majority of the genome (Table S3); however, some genes consistently had more sequence variation in multiple isolate pairs, potentially indicating enhanced recombination at these loci.

Several of these genes have been annotated with functions associated with DNA processing, transcription, repair and maintenance. This may reflect the mechanisms of recombination and horizontal gene transfer. Other genes with evidence of elevated recombination included those associated with surface exposed proteins with roles in glycosylation, motility and secretion which would form part of an initial interaction with the host/environment (Table S3). Variation in recombination rate could be influenced by differential selection pressure. The *C. jejuni* N‐acetyltransferase PseH (Cj1313) plays a key role in O‐linked glycosylation, which contributes to flagellar formation, motility and pseudaminic acid biosynthesis (McNally et al., 2006; Song, Nam, Namgung, & Yoon, 2015) and is important in host colonization (Guerry et al., 2006). The variable outer membrane protein gene *porA*, which has been used as part of extended MLST schemes (Cody, Maiden, & Dingle, 2009; Dingle, McCarthy, Cody, Peto, & Maiden, 2008) was also among those genes with evidence of elevated recombination. This may explain why weak allopatric signals have been associated with sequence variation in the *porA* gene in addition to source attribution signals (Mughini‐Gras et al., 2014; Sheppard, Colles et al., 2010; Smid et al., 2013).

Three efflux pump genes (*Cj0034c, Cj0619* and *Cj1174*) which have been implicated in fluoroquinolone resistance, showed elevated recombination and phylogeographical variation (Table S3; Ge, McDermott, White, & Meng, 2005; Luangtongkum et al., 2009). Clinical and agricultural prescription of broad‐spectrum antibiotics such as quinolones varies worldwide. Since the late 1990s, the agricultural use of fluoroquinolones has declined following governmental intervention in Europe and North America (Chang, Wang, Regev‐Yochay, Lipsitch, & Hanage, 2015; Nelson, Chiller, Powers, & Angulo, 2007); however, resistant isolates remain common and the level of resistance can vary from country to country (Pham et al., 2016). Higher levels of fluoroquinolone resistance have been observed among isolates from infected individuals who have recently returned from foreign travel (Gaudreau, Boucher, Gilbert, & Bekal, 2014). This is consistent with the higher levels of use in other parts of the world (Zhong et al., 2017). The identification of efflux pump genes among those with high levels of inferred recombination suggests that fluoroquinolone resistance provides a useful indicator for geographical segregation of isolates.

MLST‐based attribution models have been successful in assigning genomes to host reservoirs, using large test data sets (10s of thousands of isolates) to train the model. With additional isolates from other countries and appropriate source information, signatures of local recombination in *Campylobacter* genomes have the potential to identify the country of origin and attribute the source of infection among returning travellers. In this study, 74% of isolates from individuals that had declared recent foreign travel were attributed to non‐UK sources; however, in the absence of genetic elements that segregate absolutely by geography, the model relies upon the availability of large reference data sets from reservoir populations in different countries for frequency‐dependent attribution. Although this limits the applicability of the approach using currently available data the statistical genetics methodologies employed here provide a quantitative means for identifying genomic signatures of allopatry. This potentially enables the evaluation of transmission dynamics through global livestock trade networks. *Campylobacter* populations are highly structured with some lineages having greater significance in human disease than others, either because of enhanced capacity to survive through slaughter and food production (Yahara et al., 2016) or increased antimicrobial resistance (Cody, Clarke, Bowler, & Dingle, 2010; Wimalarathna et al., 2013). Monitoring the spread of these strains may be useful for evidence‐based interventions targeting strains that are a significant global health burden.

## DATA ACCESSIBILITY

Draft assembly genomes and short sequencing reads for all genomes sequenced in this study are available from the NCBI short read archive associated with BioProject: PRJNA312235 (https://www.ncbi.nlm.nih.gov/bioproject/PRJNA312235). All assembled genomes used in this study can also be downloaded together from FigShare (https://doi.org/10.6084/m9.figshare.4906634). Individual accession numbers can be found in Supplementary Table S1.

## CONFLICT OF INTEREST

The authors declare no competing interests.

## AUTHOR CONTRIBUTIONS

B.P., G.M., X.D. and S.K.S. designed research; B.P., G.M., K.Y., H.W., S.M. and X.D. performed research; B.P., G.M., K.Y., H.W., S.M., X.D., C.T.P. and S.K.S. analysed results; M.D.H., E.L.S., C.D.C., E.N.T., K.K.C., S.H., A.J.C., K.A.J., M.C.J.M., N.M. and S.K.S. provided isolates, genomes or software; and B.P., G.M., C.T.P. and S.K.S. wrote the manuscript.
