# Supplementary material for: Local genes for local bacteria: Evidence of allopatry in the genomes of transatlantic Campylobacter populations
Source: Mol Ecol. 2017 Jun 19;26(17):4497–508. doi: 10.1111/mec.14176 (PMC5600125; doi:10.1111/mec.14176)

Local genes for local bacteria: evidence of allopatry in the genomes of transatlantic *Campylobacter* populations

Ben Pascoe[1,2], Guillaume Méric[1], Koji Yahara[3,4], Helen Wimalarathna[5], Susan Murray[4], Matthew D. Hitchings[4], Emma L. Sproston[6], Catherine D. Carrillo[7], Eduardo N. Taboada[8], Kerry K. Cooper[9], Steven Huynh[10], Alison J. Cody[5], Keith A. Jolley[5], Martin C. J. Maiden[5,11], Noel D. McCarthy[5,11,12], Xavier Didelot[13], Craig T. Parker[10] and Samuel K. Sheppard[1,2,5]#

[1]The Milner Centre for Evolution, Department of Biology and Biochemistry, Bath University, Claverton Down, Bath, BA2 7AY, UK; [2]MRC CLIMB Consortium, UK; [3]Department of Bacteriology II, National Institute of Infectious Diseases, Musashimurayama, Tokyo, 208-0011, Japan; [4]Swansea University Medical School, Swansea University, Singleton Park, Swansea, SA2 8PP; [5]Department of Zoology, University of Oxford, South Parks Road, Oxford, OX1 3PS, UK; [6]Bureau of Microbial Hazards, Health Canada, 251 Sir Frederick Banting Driveway, Ottawa, K1A 0K9, Canada; [7]Canadian Food Inspection Agency, 960 Carling Avenue, Ottawa, K1A 0Y9, Canada; [8]National Microbiology Laboratory at Lethbridge, Public Health Agency of Canada, PO Box 640, Township Rd. 9-1, Lethbridge, Alberta, T1J 3Z4, Canada; [9]Department of Biology, California State University, Northridge, Northridge, California, USA; [10]Produce Safety and Microbiology Research Unit, Agricultural Research Service, US Department of Agriculture, Albany, California, USA; [11]NIHR Health Protection Research Unit in Gastrointestinal Infections, UK; [12]University of Warwick, Coventry, CV4 7AL, UK; [13]Department of Infectious Disease Epidemiology, Imperial College London, London, W2 1PG, UK

Figure S1: Neighbour-joining trees of all 57 genes showing greater than 1% diversity between pairs. Genes used in attribution model are labelled in red.

Cj0623 (hypB)

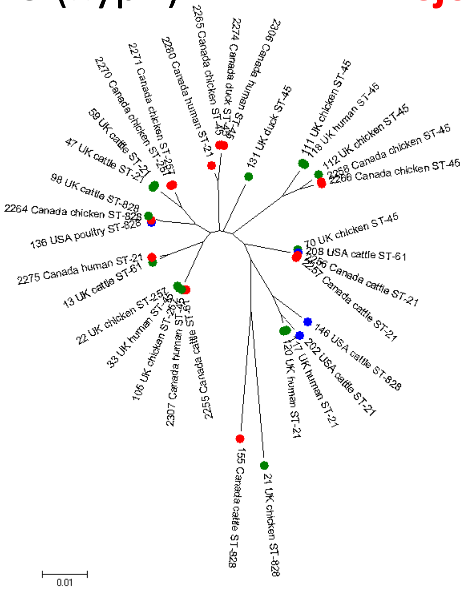

Cj0034c

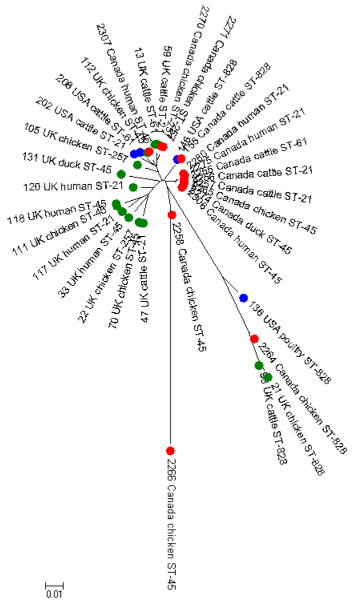

Cj0616 (pstB)

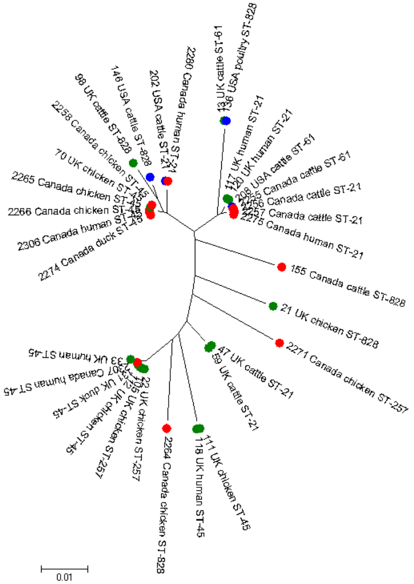

Cj0615 (pstA)

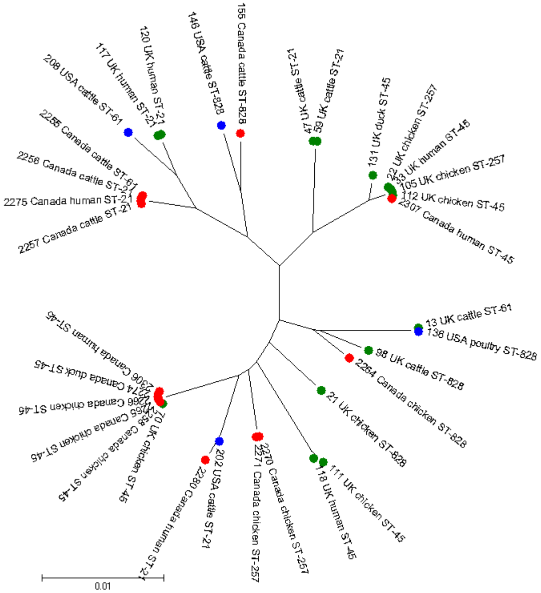

Cj0631c

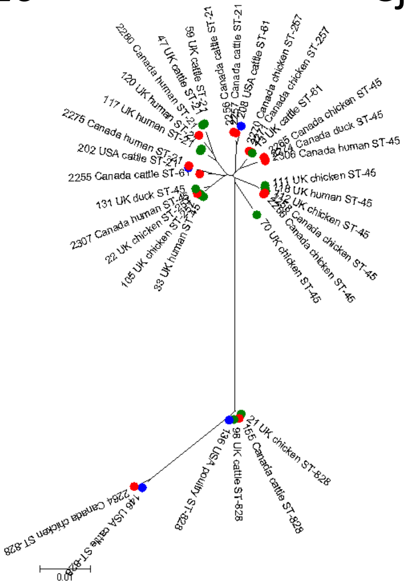

Cj0031

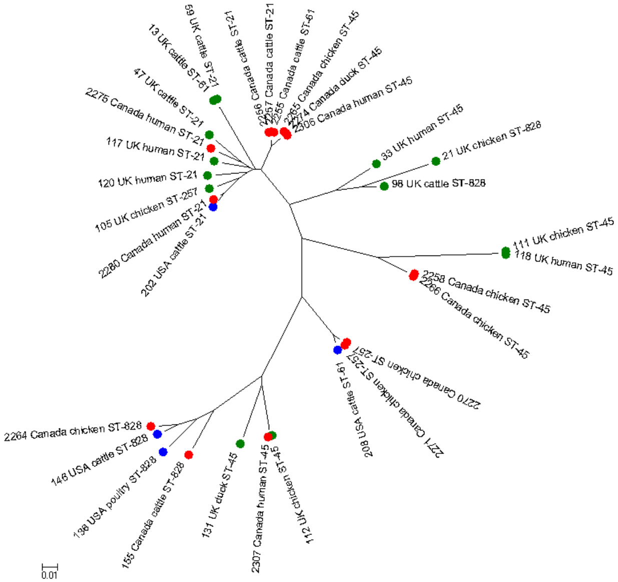

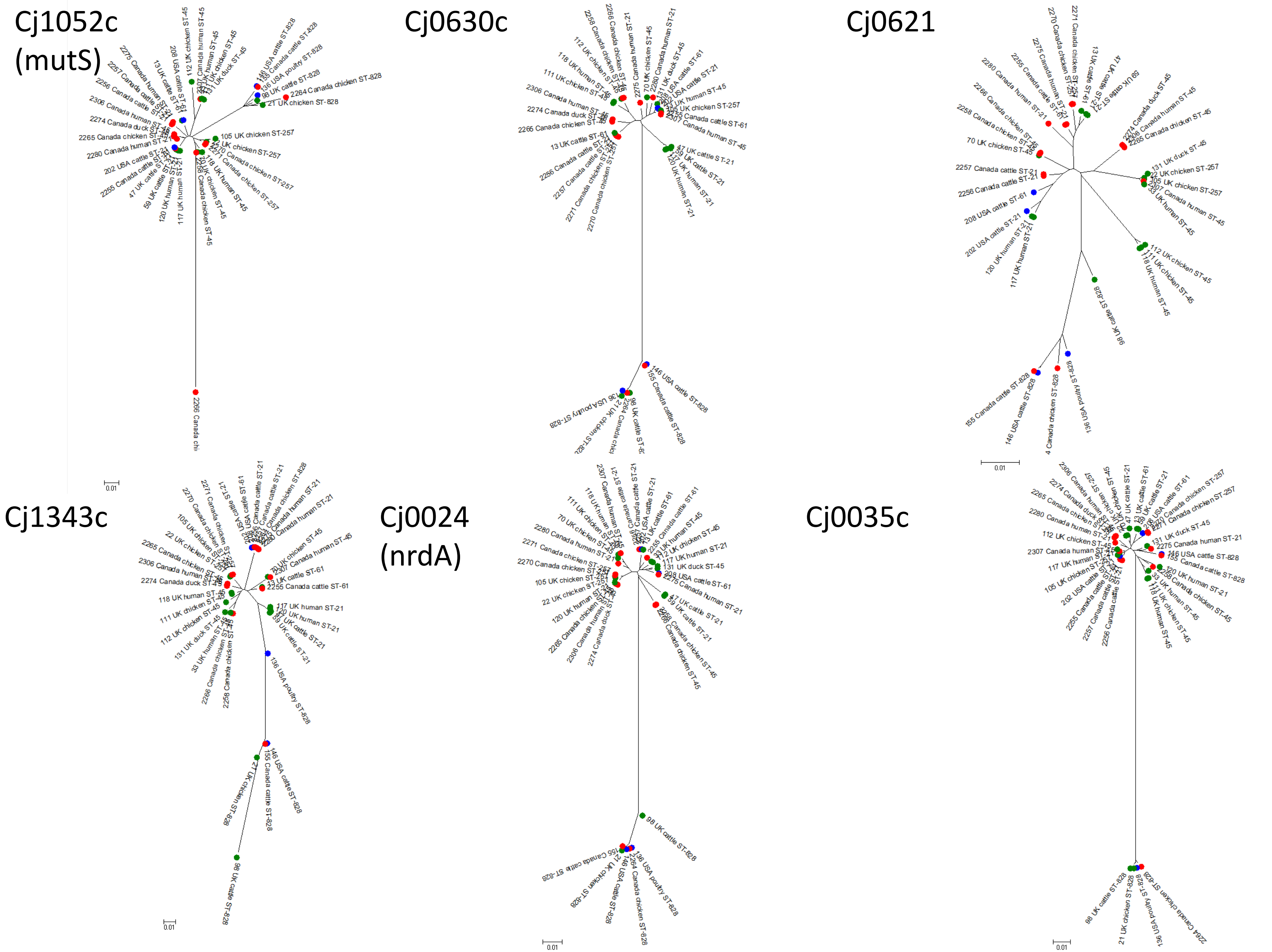
Cj1052c (mutS)
Cj0630c
Cj0621
Cj1343c
Cj0024 (nrdA)
Cj0035c
0.01

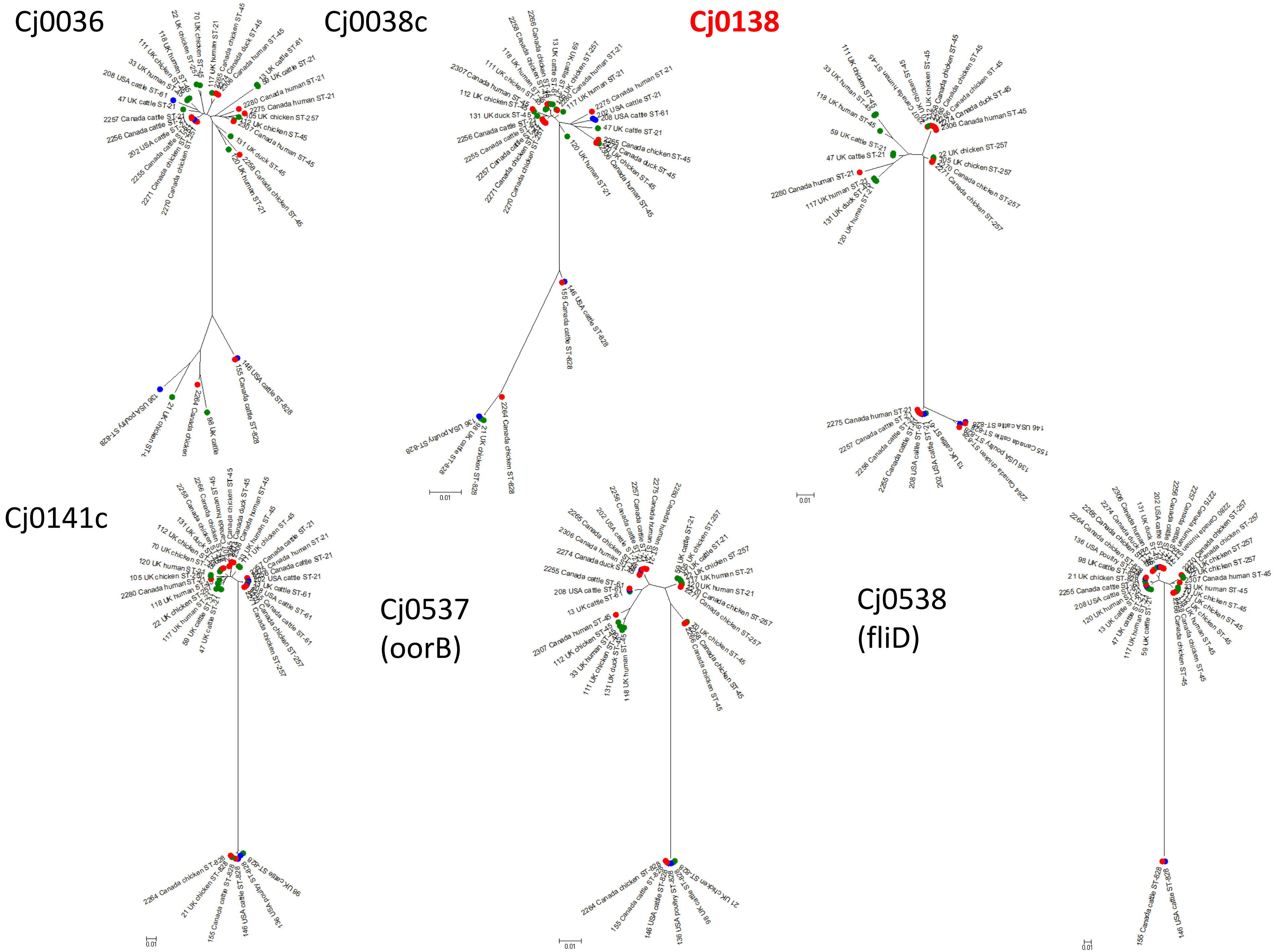
Cj0036
Cj0038c
Cj0138
Cj0141c
Cj0537
(oorB)
Cj0538
(fliD)

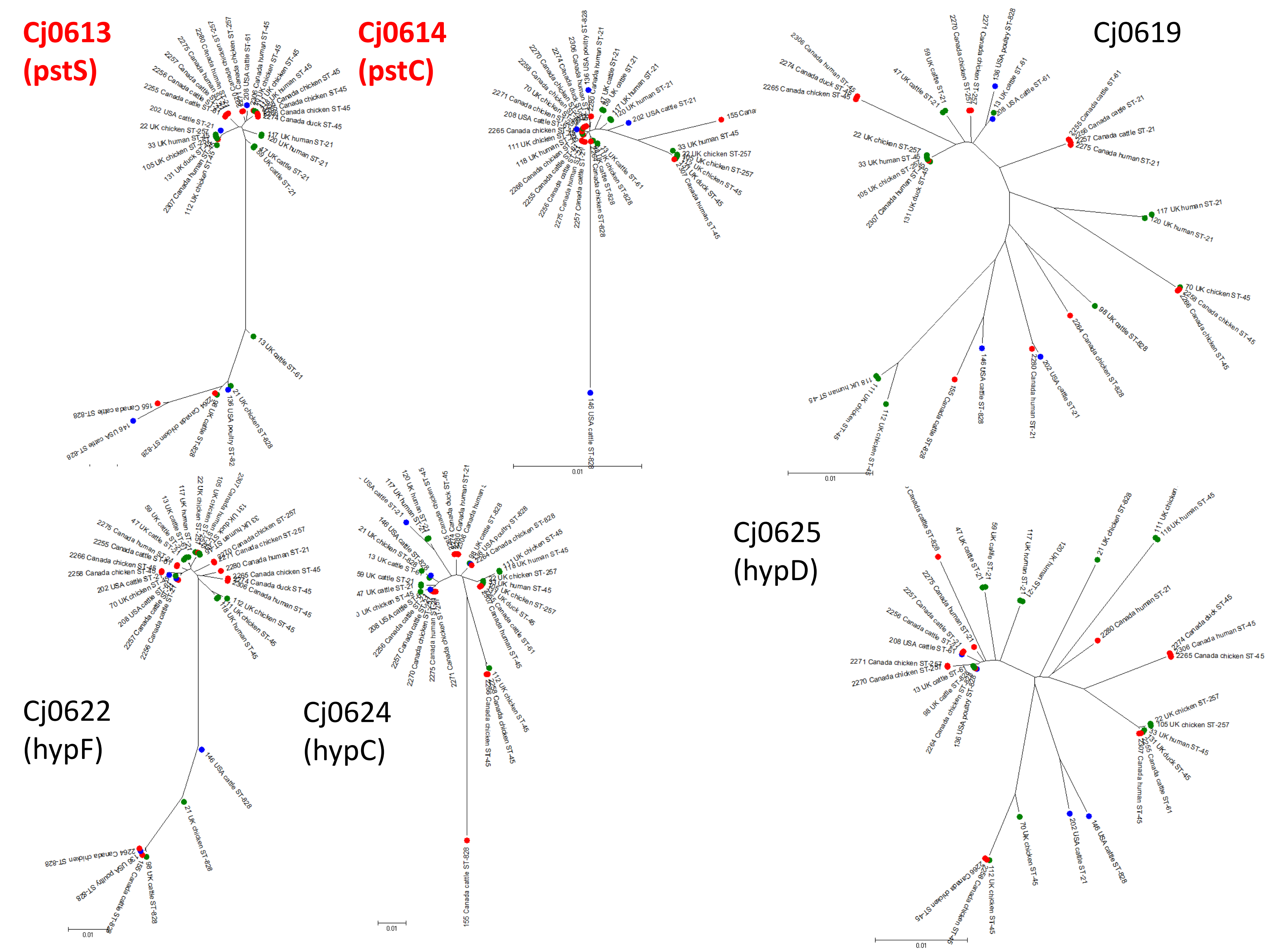
Cj0613
(pstS)
Cj0614
(pstC)
Cj0619
Cj0622
(hypF)
Cj0624
(hypC)
Cj0625
(hypD)
0.01

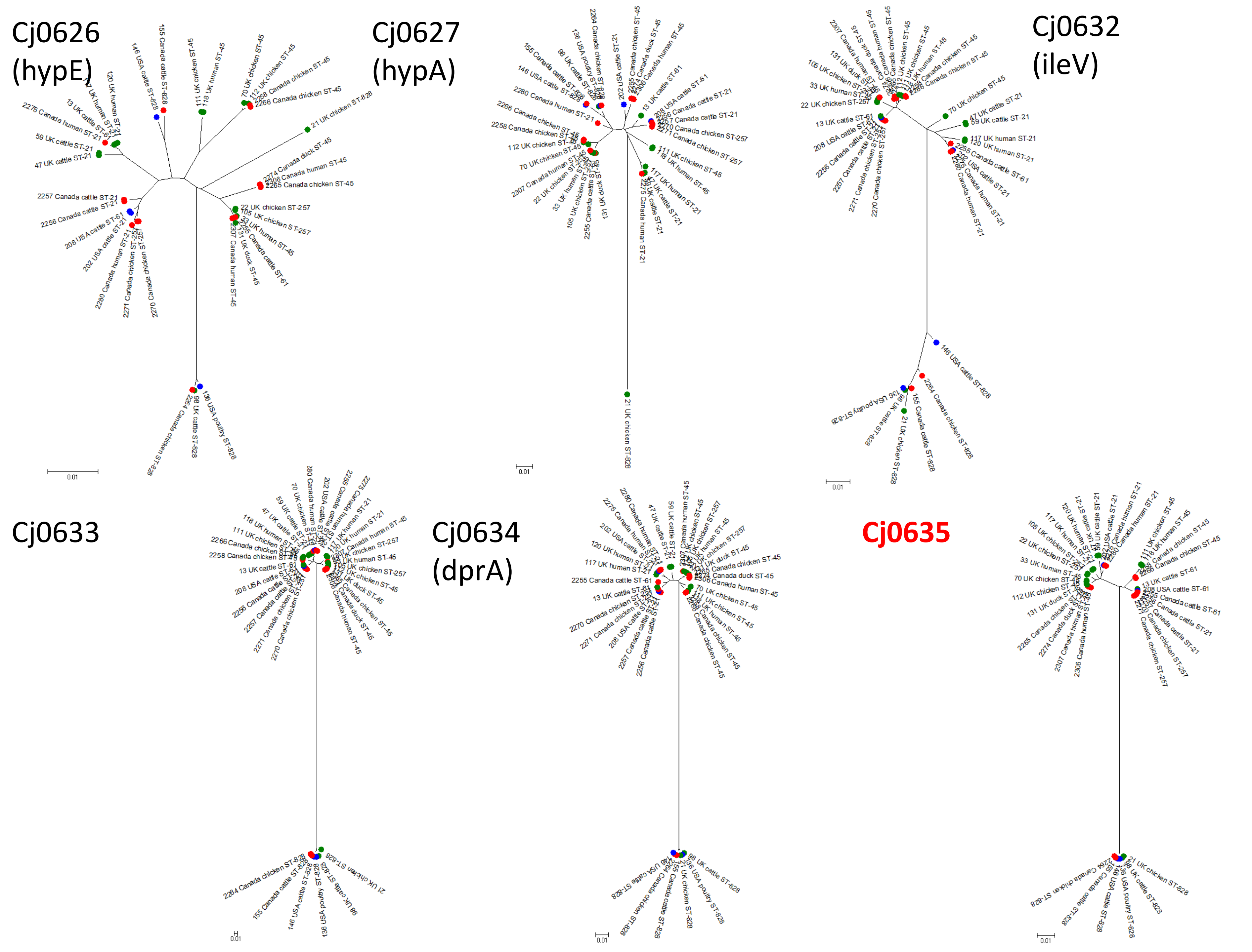
Cj0626
(hypE)
Cj0627
(hypA)
Cj0632
(ileV)
Cj0633
Cj0634
(dprA)
Cj0635
0.01

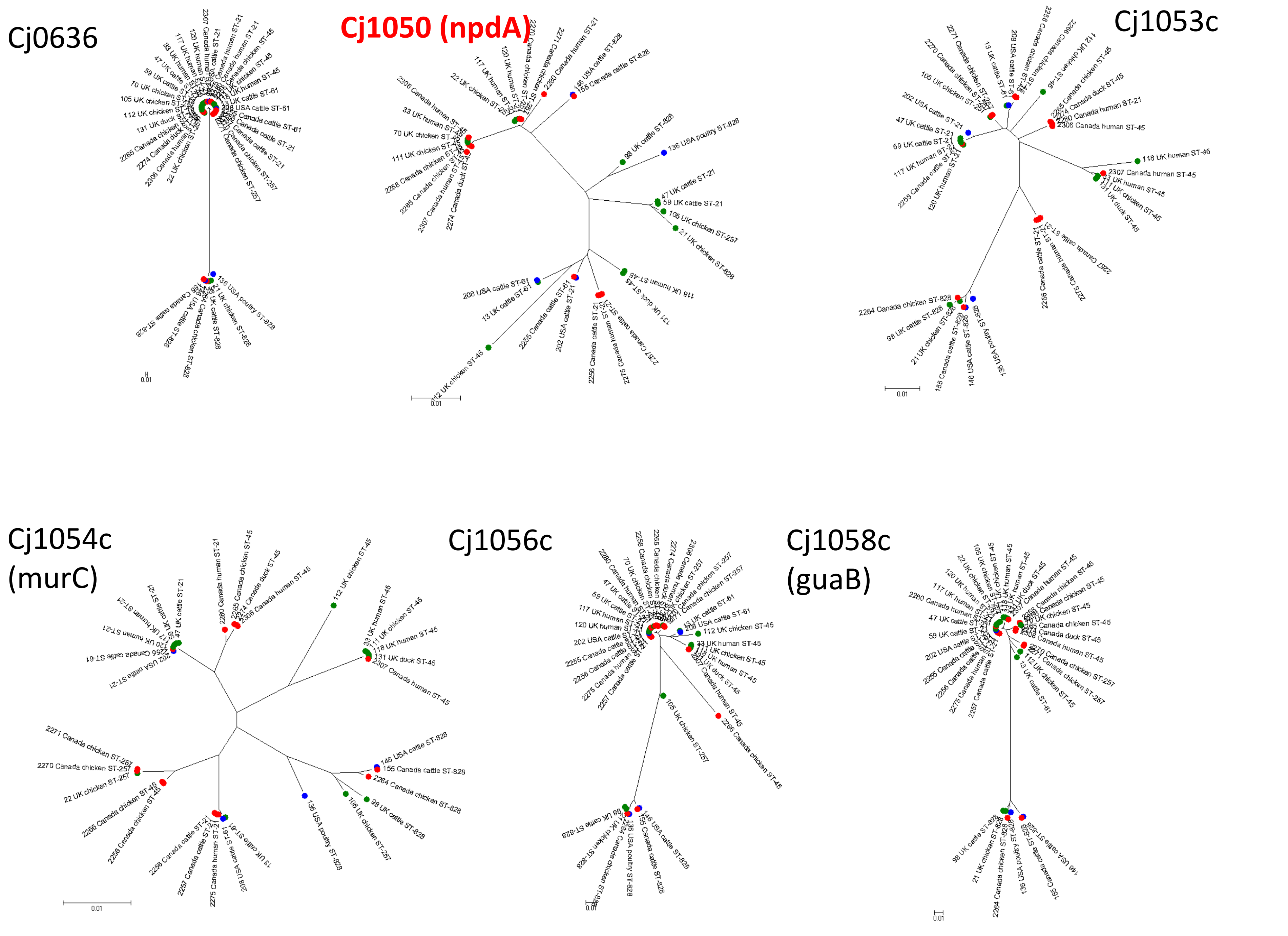
Cj0636
Cj1050 (npdA)
Cj1053c
Cj1054c (murC)
Cj1056c
Cj1058c (guaB)

Cj1066 (rdxA)

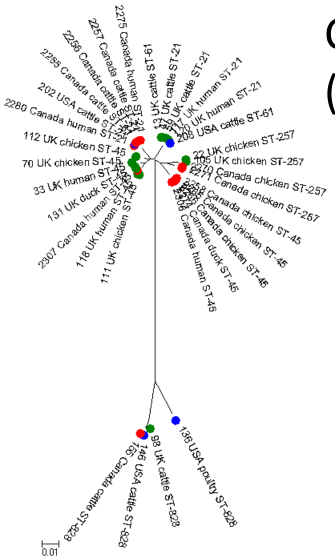

Cj1067 (pgsA)

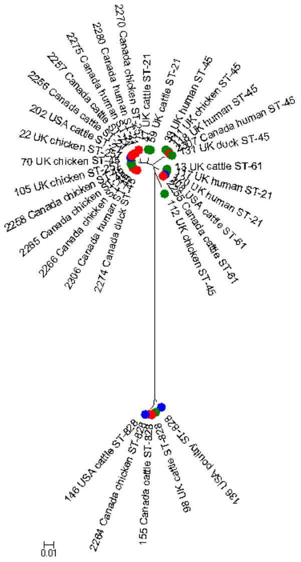

Cj1068

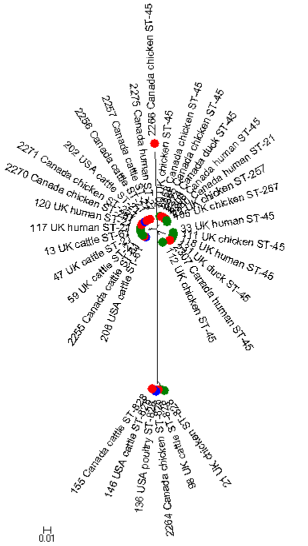

Cj1069

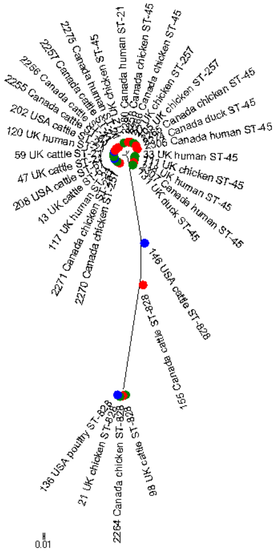

Cj1151c (hldD)

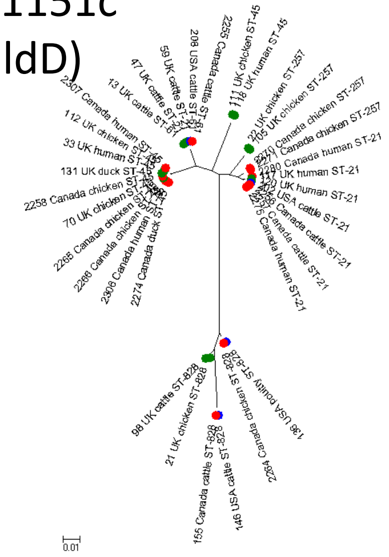

Cj1157c (dnaX)

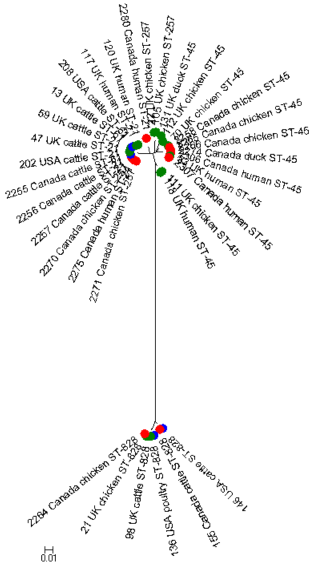

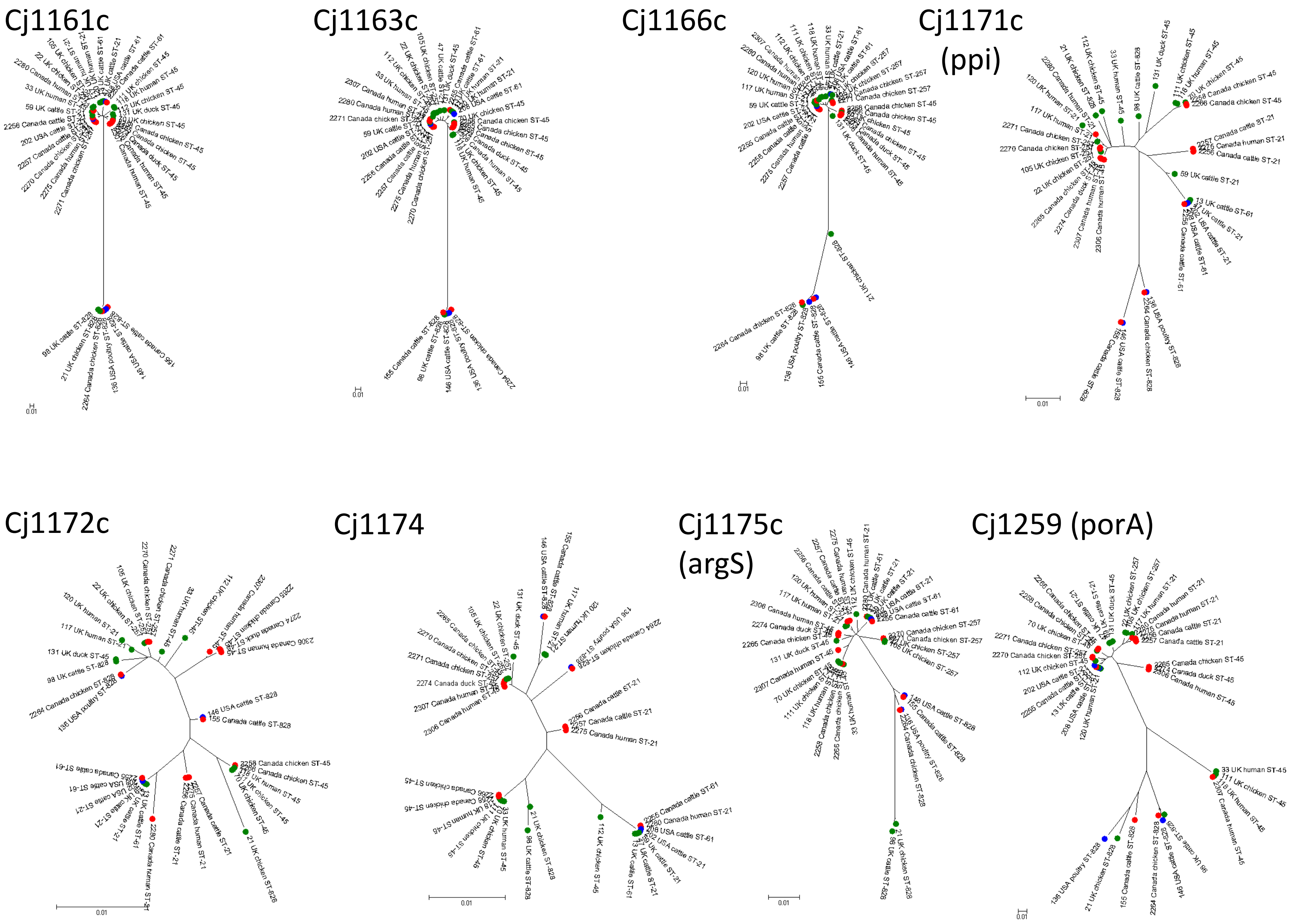
Cj1161c
Cj1163c
Cj1166c
Cj1171c (ppi)
Cj1172c
Cj1174
Cj1175c (argS)
Cj1259 (porA)
0.01

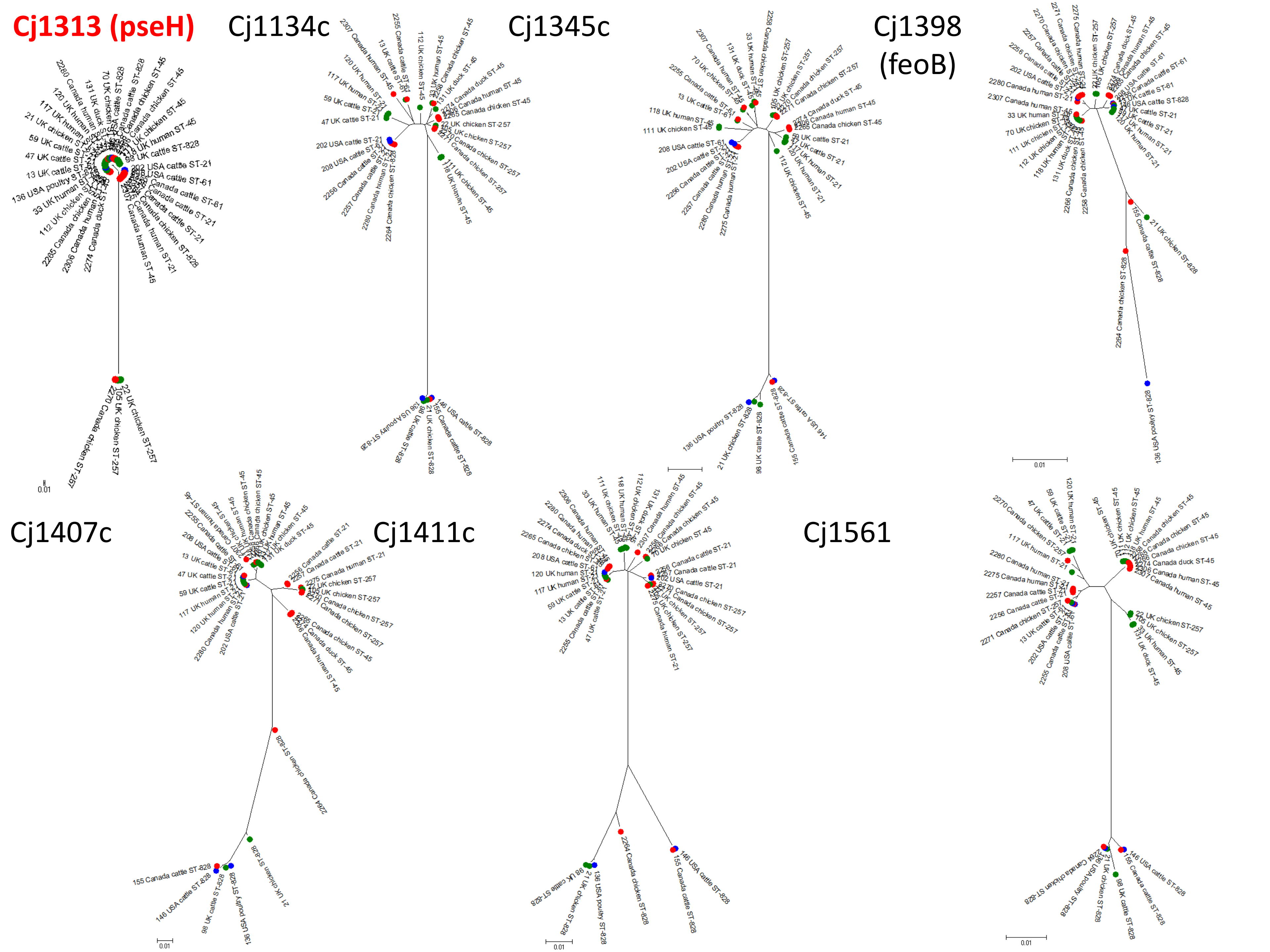
Cj1313 (pseH)
Cj1134c
Cj1345c
Cj1398 (feoB)
Cj1407c
Cj1411c
Cj1561

Figure S2: Phylogeny of 7 highly recombining epidemiological markers used to attribute biogeography using structure.

7 hyper-recombinant genes with some geographical signal

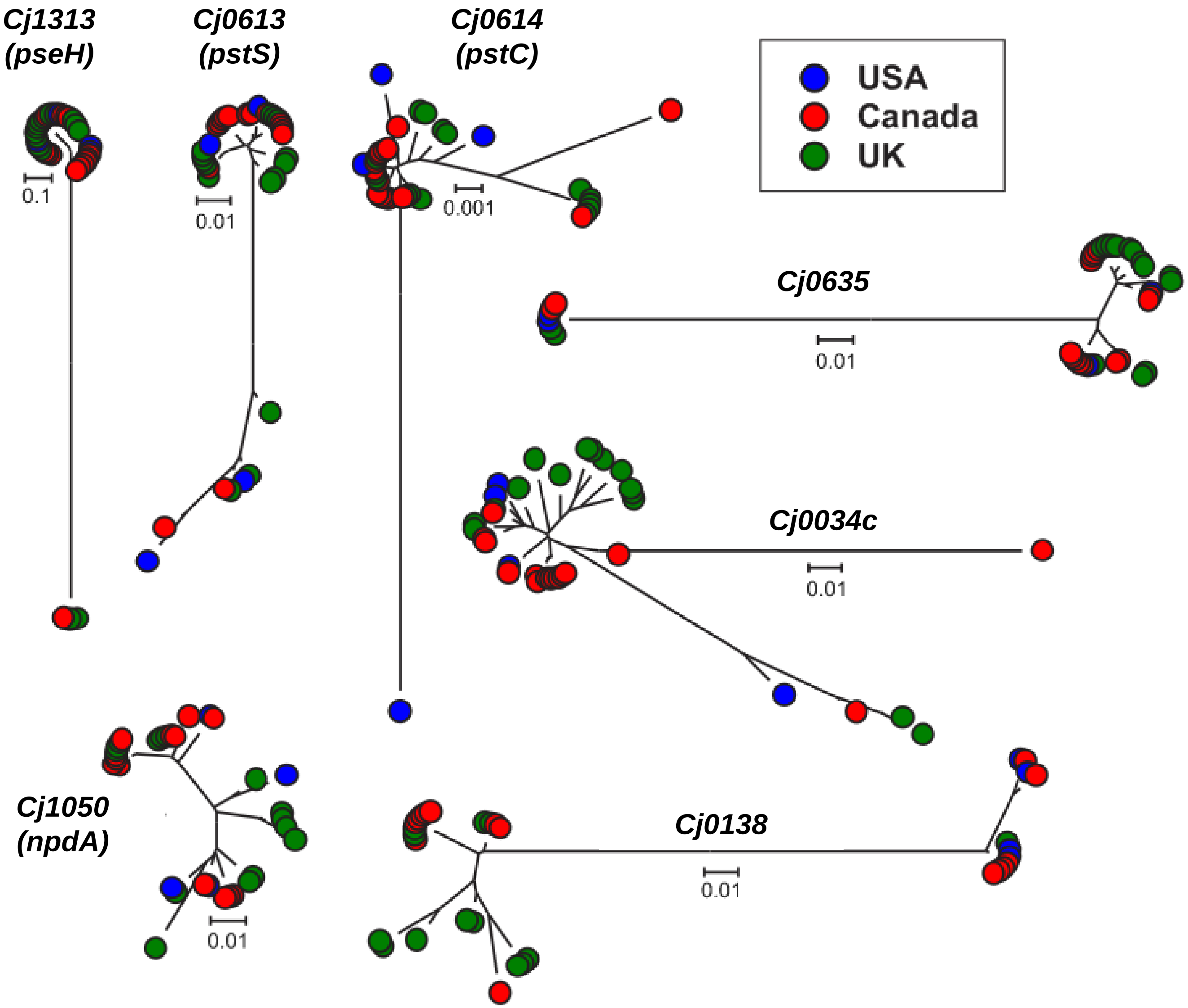

Supplement: Supplementary file 1 [file MEC-26-4497-s001.pdf]
