# Supplementary material for: Local genes for local bacteria: Evidence of allopatry in the genomes of transatlantic Campylobacter populations
Source: Mol Ecol. 2017 Jun 19;26(17):4497–508. doi: 10.1111/mec.14176 (PMC5600125; doi:10.1111/mec.14176)
Supplement: Supplementary file 2 [file MEC-26-4497-s002.pdf]

Local genes for local bacteria: evidence of allopatry in the genomes of transatlantic *Campylobacter* populations

Ben Pascoe[1,2], Guillaume Méric[1], Koji Yahara[3,4], Helen Wimalarathna[5], Susan Murray[4], Matthew D. Hitchings[4], Emma L. Sproston[6], Catherine D. Carrillo[7], Eduardo N. Taboada[8], Kerry K. Cooper[9], Steven Huynh[10], Alison J. Cody[5], Keith A. Jolley[5], Martin C. J. Maiden[5,11], Noel D. McCarthy[5,11,12], Xavier Didelot[13], Craig T. Parker[10] and Samuel K. Sheppard[1,2,5]#

[1]The Milner Centre for Evolution, Department of Biology and Biochemistry, Bath University, Claverton Down, Bath, BA2 7AY, UK; [2]MRC CLIMB Consortium, UK; [3]Department of Bacteriology II, National Institute of Infectious Diseases, Musashimurayama, Tokyo, 208-0011, Japan; [4]Swansea University Medical School, Swansea University, Singleton Park, Swansea, SA2 8PP; [5]Department of Zoology, University of Oxford, South Parks Road, Oxford, OX1 3PS, UK; [6]Bureau of Microbial Hazards, Health Canada, 251 Sir Frederick Banting Driveway, Ottawa, K1A 0K9, Canada; [7]Canadian Food Inspection Agency, 960 Carling Avenue, Ottawa, K1A 0Y9, Canada; [8]National Microbiology Laboratory at Lethbridge, Public Health Agency of Canada, PO Box 640, Township Rd. 9-1, Lethbridge, Alberta, T1J 3Z4, Canada; [9]Department of Biology, California State University, Northridge, Northridge, California, USA; [10]Produce Safety and Microbiology Research Unit, Agricultural Research Service, US Department of Agriculture, Albany, California, USA; [11]NIHR Health Protection Research Unit in Gastrointestinal Infections, UK; [12]University of Warwick, Coventry, CV4 7AL, UK; [13]Department of Infectious Disease Epidemiology, Imperial College London, London, W2 1PG, UK

Table S1: List of isolates used, including details of genome accession numbers.

Isolate ID	Isolate	Country	Year	Source	Species	CC	Pair	Project	Accession
2256	B3	Canada	2005	cattle	*Campylobacter jejuni*	ST-21	1	PRJNA312235	NFQE00000000
47	cow2674	UK	2006	cattle	*Campylobacter jejuni*	ST-21	1	ERP000129	ERR024436
2280	H14	Canada	1999	human	*Campylobacter jejuni*	ST-21	2	PRJNA312235	SAMN07158751
117	OxClina21	UK	2003	human	*Campylobacter jejuni*	ST-21	2	ERP000129	ERR024457
2271	C7	Canada	2004	chicken	*Campylobacter jejuni*	ST-257	3	PRJNA312235	NFPR00000000
22	CAMP2488	UK	2001	chicken	*Campylobacter jejuni*	ST-257	3	ERP000129	ERS007807
2274	D1	Canada	2006	duck	*Campylobacter jejuni*	ST-45	4	PRJNA312235	NFPO00000000
131	duck45	UK		duck	*Campylobacter jejuni*	ST-45	4	ERP000129	ERR024462
2258	C1	Canada	2004	chicken	*Campylobacter jejuni*	ST-45	5	PRJNA312235	NFQC00000000
112	chickd45	UK	2004	chicken	*Campylobacter jejuni*	ST-45	5	ERP000129	ERR024440
2306	H6	Canada	2008	human	*Campylobacter jejuni*	ST-45	6	PRJNA312235	NFOL00000000
33	CampsClin45	UK	2005	human	*Campylobacter jejuni*	ST-45	6	ERP000129	ERR024479
2255	B2	Canada	2004	cattle	*Campylobacter jejuni*	ST-61	7	PRJNA312235	NFQF00000000
13	CAMP61	UK	2006	cattle	*Campylobacter jejuni*	ST-61	7	ERP000129	ERS007793
2264	C15	Canada	2006	chicken	*Campylobacter coli*	ST-828	8	PRJNA312235	NFPY00000000
21	CAMP1090	UK	2003	chicken	*Campylobacter coli*	ST-828	8	ERP000129	ERS007809
2257	B4	Canada	2005	cattle	*Campylobacter jejuni*	ST-21	9	PRJNA312235	NFQD00000000
59	cow518	UK	2006	cattle	*Campylobacter jejuni*	ST-21	9	ERP000129	ERR024441
2275	H1	Canada	2000	human	*Campylobacter jejuni*	ST-21	10	PRJNA312235	NFPN00000000
120	OxClinb45	UK	2003	human	*Campylobacter jejuni*	ST-21	10	ERP000129	ERR024451
2270	C6	Canada	2004	chicken	*Campylobacter jejuni*	ST-257	11	PRJNA312235	NFPS00000000
105	chick354	UK	2004	chicken	*Campylobacter jejuni*	ST-257	11	ERP000129	ERR027222
2265	C16	Canada	2006	chicken	*Campylobacter jejuni*	ST-45	12	PRJNA312235	NFPX00000000
111	chickb45	UK	2004	chicken	*Campylobacter jejuni*	ST-45	12	ERP000129	ERR027226
2266	C2	Canada	2004	chicken	*Campylobacter jejuni*	ST-45	13	PRJNA312235	NFPW00000000
70	chickc45	UK	2009	chicken	*Campylobacter jejuni*	ST-45	13	ERP000129	ERR023266
2307	H7	Canada	unknown	human	*Campylobacter jejuni*	ST-45	14	PRJNA312235	NFOK00000000
118	OxClinb21	UK	2003	human	*Campylobacter jejuni*	ST-45	14	ERP000129	ERR024458
155	Cc1961	Canada	unknown	cattle	*Campylobacter coli*	ST-828	15	SRP001829	SRS010922
98	cow3202	UK	2003	cattle	*Campylobacter coli*	ST-828	15	ERP000129	ERR023300
151	Cc1909	Canada	unknown	cattle	*Campylobacter coli*	ST-828		SRP001829	SRS010918
153	Cc1948	Canada	unknown	cattle	*Campylobacter coli*	ST-828		SRP001829	SRS010920
154	Cc1957	Canada	unknown	cattle	*Campylobacter coli*	ST-828		SRP001829	SRS010921

159	CcLMG9854	Canada	unknown	human	*Campylobacter coli*	ST-828	SRP001829	SRS010926
166	CcLMG9860	Canada	unknown	human	*Campylobacter coli*		SRP001829	SRS010933
182	CjLMG9879	Canada	unknown	human	*Campylobacter jejuni*	ST-21	SRP001829	SRS010949
207	CjATCC43432	Canada	unknown	human	*Campylobacter jejuni*	ST-206	SRP001829	SRS010974
2248	#1	Canada	2008		*Campylobacter coli*		PRJNA312235	NFQL00000000
2249	#2	Canada	2008		*Campylobacter coli*		PRJNA312235	NFQK00000000
2250	#3	Canada	2006		*Campylobacter coli*	ST-828	PRJNA312235	NFQJ00000000
2251	#4	Canada	2006		*Campylobacter coli*	ST-828	PRJNA312235	NFQI00000000
2252	#5	Canada	2006		*Campylobacter coli*	ST-828	PRJNA312235	NFQH00000000
2253	A1	Canada	2005		*Campylobacter jejuni*	ST-45	PRJNA312235	NFQG00000000
2259	C10	Canada	2004	chicken	*Campylobacter jejuni*	ST-607	PRJNA312235	NFQB00000000
2261	C12	Canada	2004	chicken	*Campylobacter jejuni*	ST-48	PRJNA312235	NFQA00000000
2262	C13	Canada	2006	chicken	*Campylobacter jejuni*	ST-21	PRJNA312235	NFPZ00000000
2267	C3	Canada	2004	chicken	*Campylobacter jejuni*	ST-460	PRJNA312235	NFPV00000000
2268	C4	Canada	2004	chicken	*Campylobacter jejuni*	ST-460	PRJNA312235	NFPU00000000
2269	C5	Canada	2004	chicken	*Campylobacter jejuni*	ST-460	PRJNA312235	NFPT00000000
2272	C8	Canada	2004	chicken	*Campylobacter jejuni*	ST-607	PRJNA312235	NFPQ00000000
2273	C9	Canada	2004	chicken	*Campylobacter jejuni*		PRJNA312235	NFPP00000000
2276	H10	Canada	unknown	human	*Campylobacter jejuni*		PRJNA312235	NFPM00000000
2277	H11	Canada	1998	human	*Campylobacter jejuni*	ST-21	PRJNA312235	NFPL00000000
2278	H12	Canada	1998	human	*Campylobacter jejuni*		PRJNA312235	NFPK00000000
2279	H13	Canada	1999	human	*Campylobacter coli*		PRJNA312235	NFPJ00000000
2281	H15	Canada	1999	human	*Campylobacter jejuni*	ST-21	PRJNA312235	NFPI00000000
2282	H16	Canada	1999	human	*Campylobacter jejuni*	ST-206	PRJNA312235	NFPH00000000
2283	H17	Canada	2000	human	*Campylobacter jejuni*	ST-21	PRJNA312235	NFPG00000000
2284	H18	Canada	2000	human	*Campylobacter jejuni*	ST-45	PRJNA312235	NFPF00000000
2285	H19	Canada	1998	human	*Campylobacter jejuni*	ST-257	PRJNA312235	NFPE00000000
2286	H2	Canada	2000	human	*Campylobacter jejuni*	ST-21	PRJNA312235	NFPD00000000
2288	H21	Canada	2005	human	*Campylobacter jejuni*		PRJNA312235	NFPC00000000
2289	H22	Canada	2005	human	*Campylobacter jejuni*	ST-61	PRJNA312235	NFPB00000000
2290	H23	Canada	2005	human	*Campylobacter jejuni*	ST-443	PRJNA312235	NFPA00000000
2291	H24	Canada	2005	human	*Campylobacter jejuni*	ST-257	PRJNA312235	NFOZ00000000
2292	H25	Canada	2005	human	*Campylobacter jejuni*	ST-48	PRJNA312235	NFOY00000000
2293	H26	Canada	2005	human	*Campylobacter jejuni*	ST-48	PRJNA312235	NFOX00000000
2294	H27	Canada	2005	human	*Campylobacter jejuni*	ST-45	PRJNA312235	NFOW00000000
2295	H28	Canada	2005	human	*Campylobacter jejuni*		PRJNA312235	NFOV00000000

2296	H29	Canada	2005	human	*Campylobacter jejuni*	ST-21	PRJNA312235	NFOU00000000
2297	H3	Canada	2000	human	*Campylobacter jejuni*	ST-21	PRJNA312235	NFOT00000000
2298	H30	Canada	2005	human	*Campylobacter jejuni*	ST-21	PRJNA312235	NFOS00000000
2299	H31	Canada	2005	human	*Campylobacter jejuni*	ST-21	PRJNA312235	NFOR00000000
2300	H32	Canada	2005	human	*Campylobacter jejuni*		PRJNA312235	NFOQ00000000
2301	H33	Canada	2004	human	*Campylobacter jejuni*	ST-21	PRJNA312235	NFOP00000000
2302	H34	Canada	2004	human	*Campylobacter jejuni*	ST-61	PRJNA312235	NFOO00000000
2303	H35	Canada	2005	human	*Campylobacter jejuni*		PRJNA312235	NFON00000000
2304	H4	Canada	2000	human	*Campylobacter jejuni*	ST-21	PRJNA312235	NFOM00000000
2308	H8	Canada	unknown	human	*Campylobacter jejuni*		PRJNA312235	NFOJ00000000
2309	H9	Canada	unknown	human	*Campylobacter jejuni*	ST-48	PRJNA312235	NFOI00000000
2313	S1	Canada	2010	environmental	*Campylobacter jejuni*	ST-179	PRJNA312235	NFOH00000000
2314	S2	Canada	2009	environmental	*Campylobacter jejuni*	ST-45	PRJNA312235	NFOG00000000
2315	S3	Canada	2009	environmental	*Campylobacter jejuni*	ST-45	PRJNA312235	NFOF00000000
2316	S4	Canada	2010	environmental	*Campylobacter jejuni*		PRJNA312235	NFOE00000000
2317	S5	Canada	2010	environmental	*Campylobacter jejuni*	ST-45	PRJNA312235	NFOD00000000
2318	S6	Canada	2010	environmental	*Campylobacter jejuni*	ST-45	PRJNA312235	NFOC00000000
2319	U1	Canada	2008	unknown	*Campylobacter jejuni*	ST-443	PRJNA312235	NFOB00000000
2320	U2	Canada	2008	unknown	*Campylobacter jejuni*	ST-828	PRJNA312235	NFOA00000000
2321	U3	Canada	2005	unknown	*Campylobacter jejuni*	ST-45	PRJNA312235	NFNZ00000000
2322	W1	Canada	2004	environmental	*Campylobacter jejuni*		PRJNA312235	NFNY00000000
2323	W10	Canada	2004	environmental	*Campylobacter jejuni*	ST-61	PRJNA312235	NFNX00000000
2324	W11	Canada	2004	environmental	*Campylobacter jejuni*	ST-61	PRJNA312235	NFNW00000000
2325	W12	Canada	2004	environmental	*Campylobacter jejuni*	ST-257	PRJNA312235	NFNV00000000
2326	W13	Canada	2004	environmental	*Campylobacter jejuni*		PRJNA312235	NFNU00000000
2327	W14	Canada	2004	environmental	*Campylobacter jejuni*	ST-21	PRJNA312235	NFNT00000000
2328	W15	Canada	2004	environmental	*Campylobacter jejuni*	ST-21	PRJNA312235	NFNS00000000
2329	W16	Canada	2004	environmental	*Campylobacter jejuni*	ST-692	PRJNA312235	NFNR00000000
2330	W17	Canada	2004	environmental	*Campylobacter jejuni*	ST-21	PRJNA312235	NFNQ00000000
2331	W18	Canada	2005	environmental	*Campylobacter jejuni*	ST-257	PRJNA312235	NFNP00000000
2332	W19	Canada	2005	environmental	*Campylobacter jejuni*		PRJNA312235	NFNO00000000
2333	W2	Canada	2010	environmental	*Campylobacter jejuni*	ST-42	PRJNA312235	NFNN00000000
2334	W20	Canada	2006	environmental	*Campylobacter jejuni*	ST-692	PRJNA312235	NFNM00000000
2335	W21	Canada	2006	environmental	*Campylobacter jejuni*		PRJNA312235	NFNL00000000
2336	W22	Canada	2007	environmental	*Campylobacter jejuni*	ST-45	PRJNA312235	NFNK00000000
2338	W24	Canada	2007	environmental	*Campylobacter jejuni*	ST-45	PRJNA312235	NFNJ00000000

2339	W25	Canada	2007	environmental	*Campylobacter jejuni*		PRJNA312235	NFNI00000000
2341	W27	Canada	2010	environmental	*Campylobacter jejuni*	ST-283	PRJNA312235	NFNH00000000
2342	W28	Canada	2010	environmental	*Campylobacter jejuni*	ST-692	PRJNA312235	NFNG00000000
2343	W29	Canada	2010	environmental	*Campylobacter jejuni*	ST-45	PRJNA312235	NFNF00000000
2344	W3	Canada	2010	environmental	*Campylobacter jejuni*		PRJNA312235	NFNE00000000
2345	W30	Canada	2010	environmental	*Campylobacter jejuni*	ST-257	PRJNA312235	NFND00000000
2346	W31	Canada	2010	environmental	*Campylobacter jejuni*	ST-45	PRJNA312235	NFNC00000000
2347	W32	Canada	2010	environmental	*Campylobacter jejuni*	ST-283	PRJNA312235	NFNB00000000
2348	W33	Canada	2010	environmental	*Campylobacter jejuni*	ST-179	PRJNA312235	NFNA00000000
2349	W34	Canada	2010	environmental	*Campylobacter jejuni*	ST-45	PRJNA312235	NFMZ00000000
2350	W35	Canada	2010	environmental	*Campylobacter jejuni*	ST-283	PRJNA312235	NFMY00000000
2351	W36	Canada	2010	environmental	*Campylobacter jejuni*	ST-283	PRJNA312235	NFMX00000000
2352	W37	Canada	2010	environmental	*Campylobacter jejuni*	ST-283	PRJNA312235	NFMW00000000
2353	W38	Canada	2010	environmental	*Campylobacter jejuni*	ST-283	PRJNA312235	NFMV00000000
2355	W4	Canada	2010	environmental	*Campylobacter jejuni*	ST-45	PRJNA312235	NFMU00000000
2356	W40	Canada	2010	environmental	*Campylobacter jejuni*	ST-179	PRJNA312235	NFMT00000000
2357	W41	Canada	2010	environmental	*Campylobacter jejuni*	ST-42	PRJNA312235	NFMS00000000
2358	W42	Canada	2010	environmental	*Campylobacter jejuni*	ST-42	PRJNA312235	NFMR00000000
2359	W43	Canada	2010	environmental	*Campylobacter jejuni*		PRJNA312235	NFMQ00000000
2360	W44	Canada	2010	environmental	*Campylobacter jejuni*	ST-21	PRJNA312235	NFMP00000000
2361	W45	Canada	2010	environmental	*Campylobacter jejuni*	ST-42	PRJNA312235	NFMO00000000
2362	W46	Canada	2010	environmental	*Campylobacter jejuni*	ST-403	PRJNA312235	NFMN00000000
2363	W5	Canada	2010	environmental	*Campylobacter jejuni*		PRJNA312235	NFMM00000000
2364	W6	Canada	2010	environmental	*Campylobacter jejuni*		PRJNA312235	NFML00000000
2365	W7	Canada	2010	environmental	*Campylobacter jejuni*		PRJNA312235	NFMK00000000
2366	W8	Canada	2005	environmental	*Campylobacter jejuni*		PRJNA312235	NFMJ00000000
2367	W9	Canada	2010	environmental	*Campylobacter jejuni*	ST-45	PRJNA312235	NFMI00000000
2455	RM3421	Canada	1980	human	*Campylobacter jejuni*	ST-403	PRJNA312235	SRR5127254
2456	RM3414	Canada	1980	human	*Campylobacter jejuni*		PRJNA312235	NFQU00000000
2457	RM3408	Canada	1980	human	*Campylobacter jejuni*	ST-206	PRJNA312235	SRR5127252
2458	RM3407	Canada	1980	human	*Campylobacter jejuni*	ST-353	PRJNA312235	NFQT00000000
2459	RM3412	Canada	1980	human	*Campylobacter jejuni*	ST-21	PRJNA312235	NFQS00000000
2460	RM3417	Canada	1980	human	*Campylobacter jejuni*	ST-206	PRJNA312235	NFQR00000000
2461	RM1503	Canada	1980	human	*Campylobacter jejuni*	ST-508	PRJNA312235	NFQQ00000000
2462	RM3418	Canada	1980	human	*Campylobacter jejuni*	ST-353	PRJNA312235	SRR5127263
2464	RM3411	Canada	1980	human	*Campylobacter jejuni*	ST-45	PRJNA312235	NFQP00000000
2465	RM3413	Canada	1980	human	*Campylobacter jejuni*	ST-45	PRJNA312235	NFQO00000000
2466	RM3409	Canada	1980	human	*Campylobacter jejuni*	ST-48	PRJNA312235	NFQN00000000

2473	RM4777	Canada	1980	human	*Campylobacter coli*	ST-828	PRJNA312235	SRR5127259
2474	RM4784	Canada	1980	human	*Campylobacter coli*	ST-828	PRJNA312235	SRR5127262
1	182	UK	2007		*Campylobacter coli*		PRJNA177352	ANGL00000000.1
2	171	UK	2003		*Campylobacter coli*	ST-828	PRJNA177352	ANGM00000000.1
3	316	UK	2005		*Campylobacter coli*		PRJNA177352	ANGN00000000.1
4	CAMP45	UK	2005	chicken	*Campylobacter jejuni*	ST-45	PRJNA177352	ANGO00000000.1
5	CAMP2696	UK	2006	pig	*Campylobacter coli*		PRJNA177352	ANGP00000000.1
6	CAMP2681	UK	2006	chicken	*Campylobacter coli*		PRJNA177352	ANGQ00000000.1
11	CAMP2016	UK	2005	duck	*Campylobacter coli*		PRJNA177352	ANGV00000000.1
12	CAMP2326	UK	2006		*Campylobacter coli*		PRJNA177352	ANGW00000000.1
15	CAMP886	UK	unknown	pig	*Campylobacter coli*	ST-828	PRJNA177352	ANGZ00000000.1
17	CAMP2588	UK	2005	chicken	*Campylobacter coli*	ST-828	PRJNA177352	ANHB00000000.1
18	CAMP3667	UK	2004	chicken	*Campylobacter coli*	ST-1150	PRJNA177352	ANHC00000000.1
19	CAMP3129	UK	2006		*Campylobacter coli*		PRJNA177352	ANHD00000000.1
20	CAMP1487	UK	2003	chicken	*Campylobacter coli*		PRJNA177352	ANHE00000000.1
23	CAMP3311	UK	unknown	duck	*Campylobacter coli*		PRJNA177352	ANHH00000000.1
24	CAMP828	UK	unknown	chicken	*Campylobacter coli*	ST-828	PRJNA177352	ANHI00000000.1
25	CAMP3136	UK	2005		*Campylobacter coli*		PRJNA177352	ANHJ00000000.1
26	NCTC11828	UK	2007		*Campylobacter jejuni*	ST-283	PRJNA177352	CP000814.1
30	CAMP1044	UK	2007		*Campylobacter jejuni*		PRJNA177352	ANHK00000000.1
32	CampsClin11	UK	2005	human	*Campylobacter jejuni*	ST-45	ERP000129	ERR024478
34	CampsClin262	UK	2005	human	*Campylobacter jejuni*	ST-21	ERP000129	ERR024475
35	CampsClin583	UK	2005	human	*Campylobacter jejuni*	ST-45	ERP000129	ERR024481
36	CampsClin266	UK	2006	human	*Campylobacter jejuni*	ST-21	ERP000129	ERR024476
37	CampsClin883	UK	2006	human	*Campylobacter jejuni*	ST-21	ERP000129	ERR024477
39	chick2219	UK	2005	chicken	*Campylobacter jejuni*	ST-45	ERP000129	ERR024426
40	chicka21	UK	2006	chicken	*Campylobacter jejuni*	ST-21	ERP000129	ERR024431
41	cow55	UK	2006	cattle	*Campylobacter jejuni*		ERP000129	ERR024446
42	cow42	UK	2006	cattle	*Campylobacter jejuni*	ST-42	ERP000129	ERR024430
43	chick2253	UK	2006	chicken	*Campylobacter jejuni*		ERP000129	ERR024432
44	chick1717	UK	2006	chicken	*Campylobacter jejuni*		ERP000129	ERR024433
45	chick594	UK	2006	chicken	*Campylobacter jejuni*	ST-45	ERP000129	ERR024434
48	cow206	UK	2006	cattle	*Campylobacter jejuni*	ST-206	ERP000129	ERR024437
49	cow38	UK	2006	cattle	*Campylobacter jejuni*	ST-48	ERP000129	ERR024427
52	cow334	UK	2006	cattle	*Campylobacter jejuni*	ST-45	ERP000129	ERR023263
53	chicka45	UK	2006	chicken	*Campylobacter jejuni*		ERP000129	ERR023267
54	chick267	UK	2005	chicken	*Campylobacter jejuni*	ST-283	ERP000129	ERR023268
55	CampsClin230	UK	2006	human	*Campylobacter jejuni*	ST-45	ERP000129	ERR024480

56	cowa45	UK	2006	cattle	*Campylobacter jejuni*	ST-45	ERP000129	ERR023269
57	chick2213	UK	2005	chicken	*Campylobacter jejuni*	ST-45	ERP000129	ERR023270
60	CampsClin53	UK	2005	human	*Campylobacter jejuni*	ST-21	ERP000129	ERR024474
61	cow58	UK	2005	cattle	*Campylobacter jejuni*		ERP000129	ERR023272
62	cowa21	UK	2006	cattle	*Campylobacter jejuni*	ST-21	ERP000129	ERR023273
63	chickc21	UK	2006	chicken	*Campylobacter jejuni*	ST-21	ERP000129	ERR024447
64	chick25	UK	2006	chicken	*Campylobacter jejuni*	ST-661	ERP000129	ERR024448
65	chick104	UK	2006	chicken	*Campylobacter jejuni*	ST-21	ERP000129	ERR023274
66	chick353	UK	2009	chicken	*Campylobacter jejuni*	ST-353	ERP000129	ERR023264
67	chickb354	UK	2009	chicken	*Campylobacter jejuni*	ST-354	ERP000129	ERR024453
68	chick573	UK	2009	chicken	*Campylobacter jejuni*	ST-573	ERP000129	ERR023265
69	chick2568	UK	2009	chicken	*Campylobacter jejuni*	ST-661	ERP000129	ERR024456
71	chick19	UK	2009	chicken	*Campylobacter jejuni*	ST-21	ERP000129	ERR023276
72	chick50	UK	2009	chicken	*Campylobacter jejuni*	ST-21	ERP000129	ERR023280
73	chick53	UK	2009	chicken	*Campylobacter jejuni*	ST-21	ERP000129	ERR023281
74	chick262	UK	2009	chicken	*Campylobacter jejuni*	ST-21	ERP000129	ERR023282
75	chick266	UK	2009	chicken	*Campylobacter jejuni*	ST-21	ERP000129	ERR023283
77	chick1086	UK	2009	chicken	*Campylobacter jejuni*	ST-21	ERP000129	ERR023285
78	chick1360	UK	2009	chicken	*Campylobacter jejuni*	ST-21	ERP000129	ERR023286
79	chick11	UK	2009	chicken	*Campylobacter jejuni*	ST-45	ERP000129	ERR023287
80	chick137	UK	2009	chicken	*Campylobacter jejuni*	ST-257	ERP000129	ERR023277
81	chick1003	UK	2009	chicken	*Campylobacter jejuni*	ST-45	ERP000129	ERR023278
82	chick2048	UK	2009	chicken	*Campylobacter jejuni*	ST-45	ERP000129	ERR024450
83	chick2197	UK	2009	chicken	*Campylobacter jejuni*	ST-354	ERP000129	ERR023279
84	chick2223	UK	2009	chicken	*Campylobacter jejuni*	ST-45	ERP000129	ERR024452
85	cow3583	UK	2003	cattle	*Campylobacter jejuni*	ST-42	ERP000129	ERR024442
86	cow618	UK	2003	cattle	*Campylobacter jejuni*	ST-61	ERP000129	ERR023289
87	cow273	UK	2003	cattle	*Campylobacter jejuni*	ST-206	ERP000129	ERR024445
88	cow270	UK	2003	cattle	*Campylobacter jejuni*	ST-403	ERP000129	ERR023293
89	cowb21	UK	2003	cattle	*Campylobacter jejuni*	ST-21	ERP000129	ERR023294
90	cowb45	UK	2003	cattle	*Campylobacter jejuni*	ST-45	ERP000129	ERR023295
91	cowc45	UK	2003	cattle	*Campylobacter jejuni*	ST-45	ERP000129	ERR024443
92	cowd45	UK	2003	cattle	*Campylobacter jejuni*	ST-45	ERP000129	ERR024444
94	cow104	UK	2003	cattle	*Campylobacter jejuni*	ST-21	ERP000129	ERR024439
96	cow3189	UK	2003	cattle	*Campylobacter jejuni*		ERP000129	ERR023298
97	cow3201	UK	2003	cattle	*Campylobacter jejuni*	ST-21	ERP000129	ERR023299
99	cow3205	UK	2003	cattle	*Campylobacter jejuni*	ST-206	ERP000129	ERR023290
100	cow137	UK	2003	cattle	*Campylobacter jejuni*	ST-45	ERP000129	ERR023291

102	cow583	UK	2003	cattle	*Campylobacter jejuni*	ST-45	ERP000129	ERR027216
103	cow3207	UK	2003	cattle	*Campylobacter jejuni*	ST-45	ERP000129	ERR027220
104	cow3214	UK	2003	cattle	*Campylobacter jejuni*	ST-45	ERP000129	ERR027221
106	chick51	UK	2005	chicken	*Campylobacter jejuni*	ST-443	ERP000129	ERR024454
107	chick1079	UK	2004	chicken	*Campylobacter jejuni*	ST-573	ERP000129	ERR024455
108	chick574	UK	2004	chicken	*Campylobacter jejuni*	ST-574	ERP000129	ERR027223
109	chick814	UK	2004	chicken	*Campylobacter jejuni*	ST-661	ERP000129	ERR027224
110	chickb21	UK	2003	chicken	*Campylobacter jejuni*	ST-21	ERP000129	ERR027225
113	chick883	UK	2004	chicken	*Campylobacter jejuni*	ST-21	ERP000129	ERR027227
114	chick230	UK	2004	chicken	*Campylobacter jejuni*	ST-45	ERP000129	ERR027217
116	CampsClin21	UK	2005	human	*Campylobacter jejuni*		ERP000129	ERR024472
119	OxClina45	UK	2003	human	*Campylobacter jejuni*	ST-45	ERP000129	ERR024459
121	newspec	UK	2009	chicken	*Campylobacter sp.*		ERP000129	ERR027219
122	starling177	UK	unknown	starling	*Campylobacter jejuni*	ST-177	ERP000129	ERR024461
124	starling45	UK	unknown	starling	*Campylobacter jejuni*	ST-45	ERP000129	ERR024464
125	starling1020	UK	unknown	starling	*Campylobacter jejuni*	ST-682	ERP000129	ERR024465
126	goose1033	UK	unknown	goose	*Campylobacter jejuni*	ST-1034	ERP000129	ERR024466
127	goose702	UK	unknown	goose	*Campylobacter jejuni*		ERP000129	ERR024467
128	goose137	UK	unknown	goose	*Campylobacter jejuni*	ST-45	ERP000129	ERR024468
129	goose696	UK	unknown	goose	*Campylobacter jejuni*	ST-1332	ERP000129	ERR024469
130	duck702	UK	unknown	duck	*Campylobacter jejuni*	ST-702	ERP000129	ERR024470
8	CAMP1670	USA	2001	human	*Campylobacter coli*		PRJNA177352	ANGS00000000.1
9	CAMP1643	USA	2000	human	*Campylobacter coli*		PRJNA177352	ANGT00000000.1
132	Cc111-3	USA	unknown	pig	*Campylobacter coli*	ST-828	SRP001829	SRS010899
133	Cc90-3	USA	unknown	pig	*Campylobacter coli*		SRP001829	SRS010900
135	Cc2548	USA	unknown	chicken	*Campylobacter coli*		SRP001829	SRS010902
136	Cc2553	USA	unknown	chicken	*Campylobacter coli*	ST-828	SRP001829	SRS010903
137	Cc2680	USA	unknown	chicken	*Campylobacter coli*	ST-828	SRP001829	SRS010904
138	Cc2685	USA	unknown	chicken	*Campylobacter coli*	ST-828	SRP001829	SRS010905
139	Cc2688	USA	unknown	chicken	*Campylobacter coli*	ST-828	SRP001829	SRS010906
140	Cc2692	USA	unknown	chicken	*Campylobacter coli*	ST-828	SRP001829	SRS010907
141	Cc2698	USA	unknown	chicken	*Campylobacter coli*	ST-828	SRP001829	SRS010908
142	Cc84-2	USA	unknown	pig	*Campylobacter coli*	ST-828	SRP001829	SRS010909
143	Cc80352	USA	unknown	chicken	*Campylobacter coli*	ST-828	SRP001829	SRS010910
144	Cc86119	USA	unknown	chicken	*Campylobacter coli*	ST-828	SRP001829	SRS010911
145	Cc1091	USA	unknown	cattle	*Campylobacter coli*	ST-828	SRP001829	SRS010912
146	Cc1098	USA	unknown	cattle	*Campylobacter coli*	ST-828	SRP001829	SRS010913
147	Cc1148	USA	unknown	cattle	*Campylobacter coli*	ST-828	SRP001829	SRS010914

148	Cc1417	USA	unknown	cattle	*Campylobacter coli*	ST-828	SRP001829	SRS010915
149	Cc132-6	USA	unknown	pig	*Campylobacter coli*	ST-828	SRP001829	SRS010916
150	Cc1891	USA	unknown	cattle	*Campylobacter coli*	ST-828	SRP001829	SRS010917
152	Cc59-2	USA	unknown	pig	*Campylobacter coli*	ST-828	SRP001829	SRS010919
157	Cc67-8	USA	unknown	pig	*Campylobacter coli*	ST-828	SRP001829	SRS010924
164	Cc151-9	USA	unknown	pig	*Campylobacter coli*		SRP001829	SRS010931
172	Cj129-258	USA	unknown	cattle	*Campylobacter jejuni*	ST-42	SRP001829	SRS010939
173	Cj51494	USA	unknown	chicken	*Campylobacter jejuni*	ST-353	SRP001829	SRS010940
178	Cj60004	USA	unknown	chicken	*Campylobacter jejuni*		SRP001829	SRS010945
181	Cj55037	USA	unknown	chicken	*Campylobacter jejuni*	ST-45	SRP001829	SRS010948
183	Cj86605	USA	unknown	chicken	*Campylobacter jejuni*	ST-48	SRP001829	SRS010950
187	Cj53161	USA	unknown	chicken	*Campylobacter jejuni*	ST-353	SRP001829	SRS010954
193	Cj1997-1	USA	unknown	human	*Campylobacter jejuni*	ST-658	SRP001829	SRS010960
196	Cj1997-4	USA	unknown	human	*Campylobacter jejuni*	ST-48	SRP001829	SRS010963
197	Cj1997-7	USA	unknown	human	*Campylobacter jejuni*	ST-61	SRP001829	SRS010964
198	Cj1997-10	USA	unknown	human	*Campylobacter jejuni*		SRP001829	SRS010965
199	Cj1997-11	USA	unknown	human	*Campylobacter jejuni*	ST-22	SRP001829	SRS010966
200	Cj1997-14	USA	unknown	human	*Campylobacter jejuni*	ST-353	SRP001829	SRS010967
201	Cj51037	USA	unknown	chicken	*Campylobacter jejuni*	ST-353	SRP001829	SRS010968
202	Cj110-21	USA	unknown	cattle	*Campylobacter jejuni*	ST-21	SRP001829	SRS010969
203	Cj87330	USA	unknown	chicken	*Campylobacter jejuni*	ST-21	SRP001829	SRS010970
204	Cj87459	USA	unknown	chicken	*Campylobacter jejuni*	ST-353	SRP001829	SRS010971
205	Cj140-16	USA	unknown	cattle	*Campylobacter jejuni*	ST-61	SRP001829	SRS010972
206	Cj1213	USA	unknown	cattle	*Campylobacter jejuni*	ST-508	SRP001829	SRS010973
208	Cj1798	USA	unknown	cattle	*Campylobacter jejuni*	ST-61	SRP001829	SRS010975
209	Cj1854	USA	unknown	cattle	*Campylobacter jejuni*		SRP001829	SRS010976
210	Cj1893	USA	unknown	cattle	*Campylobacter jejuni*	ST-48	SRP001829	SRS010977
211	Cj1928	USA	unknown	cattle	*Campylobacter jejuni*	ST-21	SRP001829	SRS010978
2468	RM1170	USA	1980	chicken	*Campylobacter jejuni*	ST-52	PRJNA312235	NFQM00000000
2469	RM9357	USA	2012	cattle	*Campylobacter jejuni*		PRJNA312235	SRR5127255
2470	RM9168	USA	2012	cattle	*Campylobacter jejuni*	ST-21	PRJNA312235	SRR5127256
2471	RM8893	USA	2012	cattle	*Campylobacter jejuni*	ST-21	PRJNA312235	SRR5127253
2472	RM8004	USA	2012	cattle	*Campylobacter jejuni*	ST-21	PRJNA312235	SRR5127260
2475	RM10985	USA	2012	cattle	*Campylobacter jejuni*	ST-21	PRJNA312235	NFMH00000000
2476	D42a	USA	2005	chicken	*Campylobacter jejuni*	ST-21	PRJNA209346	CP007751.1
2477	M129	USA	1995	human	*Campylobacter jejuni*	ST-353	PRJNA209349	CP007749.1
2479	RM12500	USA	2012	cattle	*Campylobacter jejuni*	ST-61	PRJNA312235	SRR5127257

Table S2: List of Oxford clinical isolates used to test our biogeography attribution model. Isolate genomes and metadata downloaded from pubMLST.org/Campylobacter

id	isolate	country	year	disease	source	species	aspA	glnA	gltA	glyA	pgm	tkt	uncA	ST	clonal complex
21310	OXC5172	UK	2010	gastroenteritis	human stool	*Campylobacter coli*	33	39	30	82	113	47	17	825	ST-828 complex
21311	OXC5325	UK	2010	gastroenteritis	human stool	*Campylobacter jejuni*	7	17	2	15	23	3	12	51	ST-443 complex
21312	OXC5326	UK	2010	gastroenteritis	human stool	*Campylobacter jejuni*	8	2	5	53	11	3	1	607	ST-607 complex
21313	OXC5329	UK	2010	gastroenteritis	human stool	*Campylobacter jejuni*	3	1	5	17	11	11	6	49	ST-49 complex
21314	OXC5330	UK	2010	gastroenteritis	human stool	*Campylobacter jejuni*	8	7	4	4	125	7	1	1003	ST-45 complex
21315	OXC5331	UK	2010	gastroenteritis	human stool	*Campylobacter jejuni*	4	7	10	4	42	7	1	137	ST-45 complex
21316	OXC5332	UK	2010	gastroenteritis	human stool	*Campylobacter jejuni*	4	7	10	4	1	7	1	45	ST-45 complex
21317	OXC5333	UK	2010	gastroenteritis	human stool	*Campylobacter jejuni*	2	1	12	3	2	1	5	50	ST-21 complex
21318	OXC5334	UK	2010	gastroenteritis	human stool	*Campylobacter jejuni*	2	4	1	2	7	1	5	48	ST-48 complex
21319	OXC5335	UK	2010	gastroenteritis	human stool	*Campylobacter jejuni*	2	1	12	3	2	1	5	50	ST-21 complex
21320	OXC5336	UK	2010	gastroenteritis	human stool	*Campylobacter jejuni*	7	4	1	68	11	58	6	2131	
21321	OXC5337	UK	2010	gastroenteritis	human stool	*Campylobacter jejuni*	7	4	1	68	11	58	6	2131	
21322	OXC5338	UK	2010	gastroenteritis	human stool	*Campylobacter jejuni*		1	6	3	2	1			
21323	OXC5341	UK	2010	gastroenteritis	human stool	*Campylobacter jejuni*	10	81	50	87	120	76	52	794	ST-677 complex
21324	OXC5342	UK	2010	gastroenteritis	human stool	*Campylobacter jejuni*	24	2	2	2	10	3	1	464	ST-464 complex
21325	OXC5343	UK	2010	gastroenteritis	human stool	*Campylobacter jejuni*	4	7	10	4	10	7	1	2109	ST-45 complex
21326	OXC5344	UK	2010	gastroenteritis	human stool	*Campylobacter jejuni*	2	1	1	3	2	1	5	21	ST-21 complex
21327	OXC5346	UK	2010	gastroenteritis	human stool	*Campylobacter jejuni*	2	21	5	37	2	1	5	206	ST-206 complex
21328	OXC5348	UK	2010	gastroenteritis	human stool	*Campylobacter coli*	33	39	30	82	104	56	17	827	ST-828 complex
21329	OXC5349	UK	2010	gastroenteritis	human stool	*Campylobacter jejuni*	2	1	12	3	2	1	5	50	ST-21 complex
21330	OXC5350	UK	2010	gastroenteritis	human stool	*Campylobacter jejuni*	2	1	1	3	2	1	5	21	ST-21 complex
21331	OXC5353	UK	2010	gastroenteritis	human stool	*Campylobacter coli*	33	39	30	79	530	43	17	5149	ST-828 complex
21332	OXC5355	UK	2010	gastroenteritis	human stool	*Campylobacter jejuni*	7	17	5	10	11	3	6	1232	ST-353 complex
21333	OXC5358	UK	2010	gastroenteritis	human stool	*Campylobacter jejuni*	2	4	1	2	7	1	5	48	ST-48 complex
21334	OXC5363	UK	2010	gastroenteritis	human stool	*Campylobacter coli*	33	39	30	82	104	56	17	827	ST-828 complex
21335	OXC5364	UK	2010	gastroenteritis	human stool	*Campylobacter jejuni*	2	1	1	3	2	1	5	21	ST-21 complex
21336	OXC5368	UK	2010	gastroenteritis	human stool	*Campylobacter jejuni*	3	392	5	17	11	11	6	5483	ST-49 complex
21337	OXC5370	UK	2010	gastroenteritis	human stool	*Campylobacter coli*	33	39	30	82	104	56	17	827	ST-828 complex
21338	OXC5371	UK	2010	gastroenteritis	human stool	*Campylobacter jejuni*	24	2	2	2	10	3	1	464	ST-464 complex
21339	OXC5372	UK	2010	gastroenteritis	human stool	*Campylobacter jejuni*	2	1	5	3	2	1	5	19	ST-21 complex
21340	OXC5373	UK	2010	gastroenteritis	human stool	*Campylobacter jejuni*	2	21	5	37	60	1	5	273	ST-206 complex
21341	OXC5375	UK	2010	gastroenteritis	human stool	*Campylobacter jejuni*	24	2	2	2	10	3	1	464	ST-464 complex

21342	OXC5376	UK	2010	gastroenteritis	human stool	*Campylobacter jejuni*	9	2	4	62	4	5	6	257	ST-257 complex
21343	OXC5377	UK	2010	gastroenteritis	human stool	*Campylobacter jejuni*	2	21	5	2	59	1	5	222	ST-206 complex
21344	OXC5378	UK	2010	gastroenteritis	human stool	*Campylobacter jejuni*	2	1	21	3	2	1	5	53	ST-21 complex
21345	OXC5380	UK	2010	gastroenteritis	human stool	*Campylobacter jejuni*	9	53	2	53	11	3	3	2140	ST-574 complex
21346	OXC5381	UK	2010	gastroenteritis	human stool	*Campylobacter jejuni*	7	448	5	2	10	3	6	7176	ST-353 complex
21347	OXC5383	UK	2010	gastroenteritis	human stool	*Campylobacter jejuni*	1	3	6	4	3	3	3	22	ST-22 complex
21348	OXC5385	UK	2010	gastroenteritis	human stool	*Campylobacter jejuni*	8	10	2	2	11	12	6	354	ST-354 complex
21349	OXC5386	UK	2010	gastroenteritis	human stool	*Campylobacter coli*	33	39	30	82	113	47	17	825	ST-828 complex
21350	OXC5390	UK	2010	gastroenteritis	human stool	*Campylobacter jejuni*	9	2	4	62	4	5	6	257	ST-257 complex
21351	OXC5391	UK	2010	gastroenteritis	human stool	*Campylobacter coli*	124	39	30	82	189	47	74	2195	
21352	OXC5392	UK	2010	gastroenteritis	human stool	*Campylobacter jejuni*	1	2	3	4	5	9	3	42	ST-42 complex
21353	OXC5393	UK	2010	gastroenteritis	human stool	*Campylobacter jejuni*	2	1	12	3	2	1	5	50	ST-21 complex
21354	OXC5394	UK	2010	gastroenteritis	human stool	*Campylobacter jejuni*	7	53	2	10	11	3	3	574	ST-574 complex
21355	OXC5395	UK	2010	gastroenteritis	human stool	*Campylobacter jejuni*	9	2	4	62	4	5	6	257	ST-257 complex
21356	OXC5396	UK	2010	gastroenteritis	human stool	*Campylobacter jejuni*	1	4	2	4	185	3	6	1395	ST-658 complex
21357	OXC5397	UK	2010	gastroenteritis	human stool	*Campylobacter jejuni*	2	1	1	5	11	343	5	5242	ST-21 complex
21358	OXC5398	UK	2010	gastroenteritis	human stool	*Campylobacter jejuni*	9	2	4	62	4	5	6	257	ST-257 complex
21359	OXC5400	UK	2010	gastroenteritis	human stool	*Campylobacter jejuni*	8	10	2	2	11	12	6	354	ST-354 complex
21360	OXC5404	UK	2010	gastroenteritis	human stool	*Campylobacter jejuni*	2	15	29	48	127	99	23	5152	ST-702 complex
21361	OXC5405	UK	2010	gastroenteritis	human stool	*Campylobacter jejuni*	2	4	1	2	7	1	5	48	ST-48 complex
21362	OXC5406	UK	2010	gastroenteritis	human stool	*Campylobacter jejuni*	6	4	5	2	2	1	5	122	ST-206 complex
21363	OXC5407	UK	2010	gastroenteritis	human stool	*Campylobacter jejuni*	2	1	57	28	58	29	58	5154	ST-1332 complex
21364	OXC5410	UK	2010	gastroenteritis	human stool	*Campylobacter jejuni*	9	2	4	62	4	5	6	257	ST-257 complex
21365	OXC5411	UK	2010	gastroenteritis	human stool	*Campylobacter jejuni*	1	3	6	4	3	3	3	22	ST-22 complex
21366	OXC5412	UK	2010	gastroenteritis	human stool	*Campylobacter jejuni*	8	2	5	10	11	37	1	5134	ST-607 complex
21367	OXC5413	UK	2010	gastroenteritis	human stool	*Campylobacter jejuni*	2	1	1	3	2	1	5	21	ST-21 complex
21368	OXC5414	UK	2010	gastroenteritis	human stool	*Campylobacter jejuni*	2	1	12	3	2	1	5	50	ST-21 complex
21369	OXC5416	UK	2010	gastroenteritis	human stool	*Campylobacter jejuni*	2	1	12	3	2	1	23	3574	ST-21 complex
21370	OXC5417	UK	2010	gastroenteritis	human stool	*Campylobacter jejuni*	24	2	2	2	10	3	1	464	ST-464 complex
21371	OXC5418	UK	2010	gastroenteritis	human stool	*Campylobacter jejuni*	9	2	4	62	4	133	6	990	ST-257 complex
21372	OXC5420	UK	2010	gastroenteritis	human stool	*Campylobacter jejuni*	4	7	10	4	1	7	1	45	ST-45 complex
21373	OXC5421	UK	2010	gastroenteritis	human stool	*Campylobacter jejuni*	9	2	4	62	4	5	6	257	ST-257 complex
21374	OXC5422	UK	2010	gastroenteritis	human stool	*Campylobacter jejuni*	2	4	1	511	19	1	5	6601	ST-48 complex

21375	OXC5426	UK	2010	gastroenteritis	human stool	*Campylobacter jejuni*	7	112	42	62	11	67	6	877	
21376	OXC5427	UK	2010	gastroenteritis	human stool	*Campylobacter jejuni*	7	2	5	2	10	3	6	5	ST-353 complex
21377	OXC5428	UK	2010	gastroenteritis	human stool	*Campylobacter jejuni*	7	2	5	2	10	3	6	5	ST-353 complex
21378	OXC5431	UK	2010	gastroenteritis	human stool	*Campylobacter jejuni*	2	1	1	3	492	1	5	5018	ST-21 complex
21379	OXC5433	UK	2010	gastroenteritis	human stool	*Campylobacter jejuni*	2	4	1	2	7	1	5	48	ST-48 complex
21380	OXC5434	UK	2010	gastroenteritis	human stool	*Campylobacter jejuni*	4	7	10	4	42	7	1	137	ST-45 complex
21381	OXC5435	UK	2010	gastroenteritis	human stool	*Campylobacter jejuni*	2	1	12	3	2	1	5	50	ST-21 complex
21382	OXC5437	UK	2010	gastroenteritis	human stool	*Campylobacter jejuni*	2	17	2	3	2	1	5	883	ST-21 complex
21383	OXC5438	UK	2010	gastroenteritis	human stool	*Campylobacter jejuni*	2	1	21	3	2	1	5	53	ST-21 complex
21384	OXC5439	UK	2010	gastroenteritis	human stool	*Campylobacter jejuni*	1	4	2	2	6	3	17	61	ST-61 complex
21385	OXC5440	UK	2010	gastroenteritis	human stool	*Campylobacter jejuni*	2	4		2	7	1	5		
21386	OXC5441	UK	2010	gastroenteritis	human stool	*Campylobacter coli*	33	39	30			43	17		
21387	OXC5443	UK	2010	gastroenteritis	human stool	*Campylobacter jejuni*	1	3	6	4	3	3	3	22	ST-22 complex
21388	OXC5444	UK	2010	gastroenteritis	human stool	*Campylobacter jejuni*	2	1	5	3	2	1	5	19	ST-21 complex
21389	OXC5445	UK	2010	gastroenteritis	human stool	*Campylobacter jejuni*	2	1	12	3	2	1	5	50	ST-21 complex
21390	OXC5451	UK	2010	gastroenteritis	human stool	*Campylobacter jejuni*	2	1	12	3	2	1	5	50	ST-21 complex
21391	OXC5452	UK	2010	gastroenteritis	human stool	*Campylobacter jejuni*	7	53	2	10	11	3	3	574	ST-574 complex
21392	OXC5454	UK	2010	gastroenteritis	human stool	*Campylobacter jejuni*	2	21	5	2	59	1	5	222	ST-206 complex
21393	OXC5455	UK	2010	gastroenteritis	human stool	*Campylobacter jejuni*	62	4	5	62	2	1	5	5138	ST-206 complex
21394	OXC5456	UK	2010	gastroenteritis	human stool	*Campylobacter jejuni*	2	4	1	2	7	1	5	48	ST-48 complex
21395	OXC5457	UK	2010	gastroenteritis	human stool	*Campylobacter jejuni*	2	71	12	62	11	67	6	5142	
21396	OXC5459	UK	2010	gastroenteritis	human stool	*Campylobacter jejuni*	24	2	2	2	10	3	3	5136	ST-464 complex
21397	OXC5460	UK	2010	gastroenteritis	human stool	*Campylobacter jejuni*	24	17	2	15	23	3	12	443	ST-443 complex
21398	OXC5462	UK	2010	gastroenteritis	human stool	*Campylobacter jejuni*	2	1	12	3	2	1	5	50	ST-21 complex
21399	OXC5463	UK	2010	gastroenteritis	human stool	*Campylobacter jejuni*	2	1	5	3	2	1	5	19	ST-21 complex
21400	OXC5464	UK	2010	gastroenteritis	human stool	*Campylobacter jejuni*	8	2	5	53	11	3	1	607	ST-607 complex
21401	OXC5465	UK	2010	gastroenteritis	human stool	*Campylobacter jejuni*	2	1	1	3	2	1	5	21	ST-21 complex
21402	OXC5466	UK	2010	gastroenteritis	human stool	*Campylobacter jejuni*	8	10	2	2	11	12	6	354	ST-354 complex
21403	OXC5470	UK	2010	gastroenteritis	human stool	*Campylobacter jejuni*	8	1	6	3	2	1	1	44	ST-21 complex
21404	OXC5472	UK	2010	gastroenteritis	human stool	*Campylobacter jejuni*	7	2	5	2	10	3	6	5	ST-353 complex
21405	OXC5473	UK	2010	gastroenteritis	human stool	*Campylobacter coli*	33	39	30	82	23	43	17	6131	ST-828 complex
21406	OXC5474	UK	2010	gastroenteritis	human stool	*Campylobacter jejuni*	2	1	1	3	2	1	5	21	ST-21 complex
21407	OXC5475	UK	2010	gastroenteritis	human stool	*Campylobacter jejuni*	8	10	2	2	11	12	6	354	ST-354 complex

21408	OXC5476	UK	2010	gastroenteritis	human stool	*Campylobacter jejuni*	2	1	5	3	2	1	5	19	ST-21 complex
21409	OXC5477	UK	2010	gastroenteritis	human stool	*Campylobacter jejuni*	2	4	1	2	7	1	5	48	ST-48 complex
21410	OXC5479	UK	2010	gastroenteritis	human stool	*Campylobacter jejuni*	24	2	2	2	10	3	3	5136	ST-464 complex
21411	OXC5480	UK	2010	gastroenteritis	human stool	*Campylobacter jejuni*	7	17	5	2	86	3	1	2122	ST-353 complex
21412	OXC5627	UK	2010	gastroenteritis	human stool	*Campylobacter jejuni*	8	10	2	210	11	12	6	2033	ST-354 complex
21413	OXC5660	UK	2010	gastroenteritis	human stool	*Campylobacter jejuni*	24	21	2	2	2	59	6	2844	ST-460 complex
21414	OXC5664	UK	2010	gastroenteritis	human stool	*Campylobacter jejuni*	2	1	12	3	2	1	5	50	ST-21 complex
21415	OXC5665	UK	2010	gastroenteritis	human stool	*Campylobacter jejuni*	1	4	2	2	6	3	17	61	ST-61 complex
21416	OXC5669	UK	2010	gastroenteritis	human stool	*Campylobacter jejuni*	7	52	29	48	127	99	23	5153	ST-702 complex
21417	OXC5670	UK	2010	gastroenteritis	human stool	*Campylobacter jejuni*	24	21	2	2	2	59	6	2844	ST-460 complex
21418	OXC5671	UK	2010	gastroenteritis	human stool	*Campylobacter jejuni*	2	17	2	3	2	1	5	883	ST-21 complex
21419	OXC5672	UK	2010	gastroenteritis	human stool	*Campylobacter jejuni*	24	21	2	2	2	59	6	2844	ST-460 complex
21420	OXC5673	UK	2010	gastroenteritis	human stool	*Campylobacter jejuni*	8	10	2	2	11	12	6	354	ST-354 complex
21421	OXC5674	UK	2010	gastroenteritis	human stool	*Campylobacter jejuni*	9	25	2	10	22	3	6	52	ST-52 complex
21422	OXC5675	UK	2010	gastroenteritis	human stool	*Campylobacter jejuni*	2	4	1	2	7	1	5	48	ST-48 complex
21423	OXC5676	UK	2010	gastroenteritis	human stool	*Campylobacter jejuni*	7	17	2	15	23	3	12	51	ST-443 complex
21424	OXC5677	UK	2010	gastroenteritis	human stool	*Campylobacter coli*	33	66	30	174	188	43	17	6132	ST-828 complex
21425	OXC5679	UK	2010	gastroenteritis	human stool	*Campylobacter jejuni*	7	2	5	2	10	3	6	5	ST-353 complex
21426	OXC5680	UK	2010	gastroenteritis	human stool	*Campylobacter jejuni*	7	2	5	2	10	3	6	5	ST-353 complex
21427	OXC5681	UK	2010	gastroenteritis	human stool	*Campylobacter coli*	121	397	330	434	556	482	324	6133	
21428	OXC5682	UK	2010	gastroenteritis	human stool	*Campylobacter jejuni*	1	4	2	2	6	3	17	61	ST-61 complex
21429	OXC5685	UK	2010	gastroenteritis	human stool	*Campylobacter coli*	33	39	30	82	112	56	17	1578	ST-828 complex
21430	OXC5686	UK	2010	gastroenteritis	human stool	*Campylobacter jejuni*	8	17	5	2	10	59	6	400	ST-353 complex
21431	OXC5688	UK	2010	gastroenteritis	human stool	*Campylobacter coli*	33	39	30	82	104	56	17	827	ST-828 complex
21432	OXC5689	UK	2010	gastroenteritis	human stool	*Campylobacter jejuni*	2	4	2	4	19	3	6	658	ST-658 complex
21433	OXC5690	UK	2010	gastroenteritis	human stool	*Campylobacter jejuni*	7	2	5	2	10	3	6	5	ST-353 complex
21434	OXC5691	UK	2010	gastroenteritis	human stool	*Campylobacter jejuni*	2	1	12	3	2	1	5	50	ST-21 complex
21435	OXC5694	UK	2010	gastroenteritis	human stool	*Campylobacter jejuni*	2	4	1	2	7	1	5	48	ST-48 complex
21436	OXC5695	UK	2010	gastroenteritis	human stool	*Campylobacter jejuni*	8	10	2	2	11	12	6	354	ST-354 complex
21437	OXC5696	UK	2010	gastroenteritis	human stool	*Campylobacter jejuni*	2	1	21	3	2	1	5	53	ST-21 complex
21438	OXC5698	UK	2010	gastroenteritis	human stool	*Campylobacter jejuni*	62	4	5	2	2	1	5	572	ST-206 complex
21439	OXC5700	UK	2010	gastroenteritis	human stool	*Campylobacter jejuni*	8	10	2	2	11	12	6	354	ST-354 complex
21440	OXC5701	UK	2010	gastroenteritis	human stool	*Campylobacter jejuni*	7	17	5	2	10	3	6	353	ST-353 complex

21441	OXC5702	UK	2010	gastroenteritis	human stool	*Campylobacter jejuni*	7	17	2	15	23	3	12	51	ST-443 complex
21442	OXC5703	UK	2010	gastroenteritis	human stool	*Campylobacter jejuni*	9	2	4	62	4	5	6	257	ST-257 complex
21443	OXC5705	UK	2010	gastroenteritis	human stool	*Campylobacter coli*	33	39	30	82	113	44	17	872	ST-828 complex
21444	OXC5707	UK	2010	gastroenteritis	human stool	*Campylobacter jejuni*	2	4	1	2	7	1	5	48	ST-48 complex
21445	OXC5708	UK	2010	gastroenteritis	human stool	*Campylobacter jejuni*	2	1	42	3	148	1	5	861	ST-21 complex
21446	OXC5709	UK	2010	gastroenteritis	human stool	*Campylobacter jejuni*	9	2	4	62	4	5	12	2030	ST-257 complex
21447	OXC5710	UK	2010	gastroenteritis	human stool	*Campylobacter jejuni*	2	1	1	3	2	1	5	21	ST-21 complex
21448	OXC5711	UK	2010	gastroenteritis	human stool	*Campylobacter jejuni*	2	4	1	2	7	1	5	48	ST-48 complex
21449	OXC5712	UK	2010	gastroenteritis	human stool	*Campylobacter jejuni*	2	4	1	2	7	1	5	48	ST-48 complex
21450	OXC5713	UK	2010	gastroenteritis	human stool	*Campylobacter jejuni*	2	1	1	3	2	1	5	21	ST-21 complex
21451	OXC5715	UK	2010	gastroenteritis	human stool	*Campylobacter jejuni*	24	2	2	2	10	3	3	5136	ST-464 complex
21452	OXC5716	UK	2010	gastroenteritis	human stool	*Campylobacter jejuni*	2	4	2	2	6	1	5	38	ST-48 complex
21453	OXC5720	UK	2010	gastroenteritis	human stool	*Campylobacter jejuni*	2	1	12	3	2	1	5	50	ST-21 complex
21454	OXC5721	UK	2010	gastroenteritis	human stool	*Campylobacter jejuni*	7	2	5	2	10	3	6	5	ST-353 complex
21455	OXC5723	UK	2010	gastroenteritis	human stool	*Campylobacter coli*	33	39	30	82	104	56	17	827	ST-828 complex
21456	OXC5724	UK	2010	gastroenteritis	human stool	*Campylobacter jejuni*	2	1	5	3	2	1	5	19	ST-21 complex
21457	OXC5725	UK	2010	gastroenteritis	human stool	*Campylobacter jejuni*	2	1	12	3	2	1	5	50	ST-21 complex
21458	OXC5726	UK	2010	gastroenteritis	human stool	*Campylobacter jejuni*	7	2	5	2	10	3	6	5	ST-353 complex
21459	OXC5727	UK	2010	gastroenteritis	human stool	*Campylobacter jejuni*	24	2	2	2	10	3	1	464	ST-464 complex
21460	OXC5728	UK	2010	gastroenteritis	human stool	*Campylobacter jejuni*	2	1	5	3	2	61	5	2355	ST-21 complex
21461	OXC5731	UK	2010	gastroenteritis	human stool	*Campylobacter jejuni*	2	1	12	3	2	1	5	50	ST-21 complex
21462	OXC5732	UK	2010	gastroenteritis	human stool	*Campylobacter jejuni*	8	2	5	53	11	3	1	607	ST-607 complex
21463	OXC5733	UK	2010	gastroenteritis	human stool	*Campylobacter jejuni*	14	45	2	4	19	3	6	312	ST-658 complex
21464	OXC5734	UK	2010	gastroenteritis	human stool	*Campylobacter jejuni*	62	4	5	2	2	1	5	572	ST-206 complex
21465	OXC5737	UK	2010	gastroenteritis	human stool	*Campylobacter jejuni*	2	1	5	3	2	54	5	266	ST-21 complex
21466	OXC5739	UK	2010	gastroenteritis	human stool	*Campylobacter jejuni*	2	1	1	3	2	1	5	21	ST-21 complex
21467	OXC5740	UK	2010	gastroenteritis	human stool	*Campylobacter jejuni*	2	21	5	37	2	1	5	206	ST-206 complex
21468	OXC5741	UK	2010	gastroenteritis	human stool	*Campylobacter jejuni*	8	10	2	2	11	12	6	354	ST-354 complex
21469	OXC5742	UK	2010	gastroenteritis	human stool	*Campylobacter coli*	33	39	30	82	113	44	17	872	ST-828 complex
21470	OXC5743	UK	2010	gastroenteritis	human stool	*Campylobacter jejuni*	1	2	42	4	98	58	34	586	
21471	OXC5744	UK	2010	gastroenteritis	human stool	*Campylobacter jejuni*	4	7	10	4	42	7	1	137	ST-45 complex
21472	OXC5745	UK	2010	gastroenteritis	human stool	*Campylobacter jejuni*	2	4	1	2	7	1	5	48	ST-48 complex
21473	OXC5747	UK	2010	gastroenteritis	human stool	*Campylobacter jejuni*	9	25	2	10	23	3	6	775	ST-52 complex

21474	OXC5748	UK	2010	gastroenteritis	human stool	*Campylobacter jejuni*	9	25	2	10	23	3	6	775	ST-52 complex
21475	OXC5750	UK	2010	gastroenteritis	human stool	*Campylobacter jejuni*	8	17	5	2	10	59	6	400	ST-353 complex
21476	OXC5751	UK	2010	gastroenteritis	human stool	*Campylobacter jejuni*	9	25	2	10	23	3	6	775	ST-52 complex
21477	OXC5753	UK	2010	gastroenteritis	human stool	*Campylobacter jejuni*	24	30	1	2	89	3	6	5144	ST-460 complex
21478	OXC5754	UK	2010	gastroenteritis	human stool	*Campylobacter jejuni*	2	1	1	3	2	1	5	21	ST-21 complex
21479	OXC5757	UK	2010	gastroenteritis	human stool	*Campylobacter jejuni*	2	1	1	3	2	1	5	21	ST-21 complex
21480	OXC5758	UK	2010	gastroenteritis	human stool	*Campylobacter jejuni*	7	17	2	15	23	3	12	51	ST-443 complex
21481	OXC5759	UK	2010	gastroenteritis	human stool	*Campylobacter jejuni*	24	2	5	72	2	5	6	1374	
21482	OXC5761	UK	2010	gastroenteritis	human stool	*Campylobacter jejuni*	2	4	1	93	11	3	6	523	ST-658 complex
21483	OXC5762	UK	2011	gastroenteritis	human stool	*Campylobacter jejuni*	1	6	60	24	12	28	1	508	ST-508 complex
21484	OXC5763	UK	2011	gastroenteritis	human stool	*Campylobacter coli*	33	39	30	82	113	47	17	825	ST-828 complex
21485	OXC5764	UK	2011	gastroenteritis	human stool	*Campylobacter jejuni*	7	17	2	15	23	3	12	51	ST-443 complex
21486	OXC5765	UK	2011	gastroenteritis	human stool	*Campylobacter jejuni*	24	2	2	2	10	3	1	464	ST-464 complex
21487	OXC5766	UK	2011	gastroenteritis	human stool	*Campylobacter jejuni*	2	1	12	3	2	1	5	50	ST-21 complex
21488	OXC5767	UK	2011	gastroenteritis	human stool	*Campylobacter jejuni*	2	1	21	3	2	1	5	53	ST-21 complex
21489	OXC5768	UK	2011	gastroenteritis	human stool	*Campylobacter coli*	292	66	30	82	113	206	17	5150	
21490	OXC5769	UK	2011	gastroenteritis	human stool	*Campylobacter jejuni*	6	4	5	2	2	456	5	6134	
21491	OXC5771	UK	2011	gastroenteritis	human stool	*Campylobacter jejuni*	2	1	12	3	2	1	5	50	ST-21 complex
21492	OXC5772	UK	2011	gastroenteritis	human stool	*Campylobacter jejuni*	8	1	6	3	2	1	12	2135	ST-21 complex
21493	OXC5773	UK	2011	gastroenteritis	human stool	*Campylobacter coli*	33	39	30	82	104	43	17	828	ST-828 complex
21494	OXC5774	UK	2011	gastroenteritis	human stool	*Campylobacter jejuni*	2	1	5	3	2	3	5	190	ST-21 complex
21495	OXC5775	UK	2011	gastroenteritis	human stool	*Campylobacter jejuni*	2	4	2	4	19	3	6	658	ST-658 complex
21496	OXC5776	UK	2011	gastroenteritis	human stool	*Campylobacter jejuni*	7	53	2	10	11	3	3	574	ST-574 complex
21497	OXC5777	UK	2011	gastroenteritis	human stool	*Campylobacter jejuni*	24	2	2	2	10	3	1	464	ST-464 complex
21498	OXC5778	UK	2011	gastroenteritis	human stool	*Campylobacter jejuni*	22	15	4	64	74	25	23	1709	ST-1034 complex
21499	OXC5779	UK	2011	gastroenteritis	human stool	*Campylobacter jejuni*	9	2	4	62	4	5	6	257	ST-257 complex
21500	OXC5780	UK	2011	gastroenteritis	human stool	*Campylobacter jejuni*	7	2	5	2	10	3	6	5	ST-353 complex
21501	OXC5781	UK	2011	gastroenteritis	human stool	*Campylobacter jejuni*	9	2	4	62	4	5	6	257	ST-257 complex
23492	OXC5482	UK	2010	gastroenteritis	human stool	*Campylobacter coli*	33	39	65	79	104	85	17	2273	ST-828 complex
23493	OXC5484	UK	2010	gastroenteritis	human stool	*Campylobacter jejuni*	2	4	1	2	7	1	5	48	ST-48 complex
23494	OXC5486	UK	2010	gastroenteritis	human stool	*Campylobacter coli*	33	153	122	82	113	44	17	5158	
23495	OXC5490	UK	2010	gastroenteritis	human stool	*Campylobacter jejuni*	22	61	4	64	74	25	23	977	ST-1034 complex
23496	OXC5493	UK	2010	gastroenteritis	human stool	*Campylobacter jejuni*	1	4	2	2	6	3	17	61	ST-61 complex

23497	OXC5495	UK	2010	gastroenteritis	human stool	*Campylobacter jejuni*	2	1	1	3	2	1	5	21	ST-21 complex
23498	OXC5497	UK	2010	gastroenteritis	human stool	*Campylobacter jejuni*	9	2	4	62	4	5	6	257	ST-257 complex
23499	OXC5498	UK	2010	gastroenteritis	human stool	*Campylobacter jejuni*	2	1	1	3	2	1	5	21	ST-21 complex
23500	OXC5499	UK	2010	gastroenteritis	human stool	*Campylobacter jejuni*	7	2	5	2	10	3	6	5	ST-353 complex
23501	OXC5604	UK	2010	gastroenteritis	human stool	*Campylobacter jejuni*	8	10	2	210	11	12	6	2033	ST-354 complex
23502	OXC5605	UK	2010	gastroenteritis	human stool	*Campylobacter coli*	33	39	30	79	113	43	17	832	ST-828 complex
23503	OXC5608	UK	2010	gastroenteritis	human stool	*Campylobacter jejuni*	9	25	2	10	22	3	6	52	ST-52 complex
23504	OXC5613	UK	2010	gastroenteritis	human stool	*Campylobacter jejuni*	1	4	2	2	6	3	17	61	ST-61 complex
23505	OXC5614	UK	2010	gastroenteritis	human stool	*Campylobacter jejuni*	2	4	1	4	19	62	5	475	ST-48 complex
23506	OXC5616	UK	2010	gastroenteritis	human stool	*Campylobacter jejuni*	7	53	2	10	11	3	3	574	ST-574 complex
23507	OXC5617	UK	2010	gastroenteritis	human stool	*Campylobacter jejuni*	2	1	21	3	2	1	5	53	ST-21 complex
23508	OXC5618	UK	2010	gastroenteritis	human stool	*Campylobacter jejuni*	2	1	1	3	2	1	5	21	ST-21 complex
23509	OXC5620	UK	2010	gastroenteritis	human stool	*Campylobacter jejuni*	7	17	2	15	23	3	12	51	ST-443 complex
23510	OXC5622	UK	2010	gastroenteritis	human stool	*Campylobacter jejuni*	7	71	5	190	11	67	1	1526	
23511	OXC5623	UK	2010	gastroenteritis	human stool	*Campylobacter jejuni*	8	1	6	3	2	1	384	7172	ST-21 complex
23512	OXC5625	UK	2010	gastroenteritis	human stool	*Campylobacter jejuni*	2	1	1	3	2	1	5	21	ST-21 complex
23513	OXC5626	UK	2010	gastroenteritis	human stool	*Campylobacter jejuni*	2	4	1	4	19	62	5	475	ST-48 complex
23514	OXC5628	UK	2010	gastroenteritis	human stool	*Campylobacter jejuni*	2	1	12	3	2	1	5	50	ST-21 complex
23515	OXC5629	UK	2010	gastroenteritis	human stool	*Campylobacter coli*	33	39	30	79	104	35	17	855	ST-828 complex
23516	OXC5630	UK	2010	gastroenteritis	human stool	*Campylobacter jejuni*	24	2	2	2	10	289	1	2315	ST-464 complex
23517	OXC5631	UK	2010	gastroenteritis	human stool	*Campylobacter jejuni*	24	2	2	2	10	289	1	2315	ST-464 complex
23518	OXC5632	UK	2010	gastroenteritis	human stool	*Campylobacter jejuni*	9	2	4	62	4	5	6	257	ST-257 complex
23519	OXC5633	UK	2010	gastroenteritis	human stool	*Campylobacter jejuni*	7	17	5	2	86	232	1	2076	ST-353 complex
23520	OXC5634	UK	2010	gastroenteritis	human stool	*Campylobacter jejuni*	7	2	5	2	10	3	6	5	ST-353 complex
23521	OXC5635	UK	2010	gastroenteritis	human stool	*Campylobacter coli*	124	39	30	79	104	47	17	1541	ST-828 complex
23522	OXC5636	UK	2010	gastroenteritis	human stool	*Campylobacter jejuni*	2	4	1	2	7	1	5	48	ST-48 complex
23523	OXC5638	UK	2010	gastroenteritis	human stool	*Campylobacter jejuni*	7	15	5	62	67	3	6	581	ST-353 complex
23524	OXC5639	UK	2010	gastroenteritis	human stool	*Campylobacter jejuni*	2	17	2	3	2	1	5	883	ST-21 complex
23525	OXC5640	UK	2010	gastroenteritis	human stool	*Campylobacter jejuni*	2	4	1	2	7	1	5	48	ST-48 complex
23526	OXC5641	UK	2010	gastroenteritis	human stool	*Campylobacter jejuni*	9	2	4	62	4	5	12	2030	ST-257 complex
23527	OXC5642	UK	2010	gastroenteritis	human stool	*Campylobacter jejuni*	7	71	2	62	11	34	6	5244	
23528	OXC5643	UK	2010	gastroenteritis	human stool	*Campylobacter jejuni*	9	2	4	62	4	5	6	257	ST-257 complex
23529	OXC5645	UK	2010	gastroenteritis	human stool	*Campylobacter jejuni*	24	2	2	2	10	3	1	464	ST-464 complex

23530	OXC5646	UK	2010	gastroenteritis	human stool	*Campylobacter jejuni*	7	17	2	15	23	3	12	51	ST-443 complex
23531	OXC5647	UK	2010	gastroenteritis	human stool	*Campylobacter jejuni*	2	1	5	3	2	1	5	19	ST-21 complex
23532	OXC5648	UK	2010	gastroenteritis	human stool	*Campylobacter jejuni*	62		5	2	2	1	5		
23533	OXC5649	UK	2010	gastroenteritis	human stool	*Campylobacter jejuni*	2	1	1	3	2	1	5	21	ST-21 complex
23534	OXC5651	UK	2010	gastroenteritis	human stool	*Campylobacter jejuni*	2	1	12	3	2	1	5	50	ST-21 complex
23535	OXC5652	UK	2010	gastroenteritis	human stool	*Campylobacter jejuni*	2	1	1	3	2	1	5	21	ST-21 complex
23536	OXC5782	UK	2011	gastroenteritis	human stool	*Campylobacter jejuni*	7	53	2	10	11	3	3	574	ST-574 complex
23537	OXC5785	UK	2011	gastroenteritis	human stool	*Campylobacter jejuni*	1	2	3	4	5	9	3	42	ST-42 complex
23538	OXC5786	UK	2011	gastroenteritis	human stool	*Campylobacter jejuni*	7	17	5	2	86	3	1	2122	ST-353 complex
23539	OXC5787	UK	2011	gastroenteritis	human stool	*Campylobacter jejuni*	7	53	2	10	11	3	3	574	ST-574 complex
23540	OXC5788	UK	2011	gastroenteritis	human stool	*Campylobacter jejuni*	9	2	4	62	4	5	6	257	ST-257 complex
23541	OXC5789	UK	2011	gastroenteritis	human stool	*Campylobacter jejuni*	24	2	5	53	23	3	1	904	ST-607 complex
23542	OXC5791	UK	2011	gastroenteritis	human stool	*Campylobacter jejuni*	2	1	1	3	2	1	5	21	ST-21 complex
23543	OXC5792	UK	2011	gastroenteritis	human stool	*Campylobacter jejuni*	7	53	2	10	11	3	3	574	ST-574 complex
23544	OXC5793	UK	2011	gastroenteritis	human stool	*Campylobacter jejuni*	2	4	1	93	11	3	6	523	ST-658 complex
23545	OXC5795	UK	2011	gastroenteritis	human stool	*Campylobacter jejuni*	7	28	4	28	17	34	12	573	ST-573 complex
23546	OXC5796	UK	2011	gastroenteritis	human stool	*Campylobacter coli*	33	39	30	82	113	44	17	872	ST-828 complex
23547	OXC5797	UK	2011	gastroenteritis	human stool	*Campylobacter jejuni*	8	10	2	210	11	12	6	2033	ST-354 complex
23548	OXC5798	UK	2011	gastroenteritis	human stool	*Campylobacter jejuni*	7	1	2	83	2	3	6	441	
23549	OXC5799	UK	2011	gastroenteritis	human stool	*Campylobacter jejuni*	2	1	12	3	2	1	5	50	ST-21 complex
23550	OXC5800	UK	2011	gastroenteritis	human stool	*Campylobacter jejuni*	22	15	4	64	74	25	23	1709	ST-1034 complex
23551	OXC5801	UK	2011	gastroenteritis	human stool	*Campylobacter jejuni*	7	17	2	15	23	3	12	51	ST-443 complex
23552	OXC5802	UK	2011	gastroenteritis	human stool	*Campylobacter jejuni*	8	10	5	2	11	12	6	1038	ST-354 complex
23553	OXC5803	UK	2011	gastroenteritis	human stool	*Campylobacter jejuni*	9		2	10	22	3	6		
23554	OXC5805	UK	2011	gastroenteritis	human stool	*Campylobacter jejuni*	7	17	2	15	23	3	12	51	ST-443 complex
23555	OXC5807	UK	2011	gastroenteritis	human stool	*Campylobacter jejuni*	24	2	2	2	10	3	3	5136	ST-464 complex
23556	OXC5808	UK	2011	gastroenteritis	human stool	*Campylobacter jejuni*	2	1	21	3	2	1	5	53	ST-21 complex
23557	OXC5809	UK	2011	gastroenteritis	human stool	*Campylobacter jejuni*	7	17	2	15	23	3	12	51	ST-443 complex
23558	OXC5810	UK	2011	gastroenteritis	human stool	*Campylobacter coli*	33	39	30	82	189	44	17	5165	ST-828 complex
23559	OXC5811	UK	2011	gastroenteritis	human stool	*Campylobacter jejuni*	8	1	6	3	2	1	12	2135	ST-21 complex
23560	OXC5812	UK	2011	gastroenteritis	human stool	*Campylobacter jejuni*	7	53	2	10	11	3	3	574	ST-574 complex
23561	OXC5813	UK	2011	gastroenteritis	human stool	*Campylobacter jejuni*	7	2	5	2	10	3	6	5	ST-353 complex
23562	OXC5814	UK	2011	gastroenteritis	human stool	*Campylobacter jejuni*	2	4	1	2	7	1	5	48	ST-48 complex

23563	OXC5815	UK	2011	gastroenteritis	human stool	*Campylobacter jejuni*	7	53	2	10	11	3	3	574	ST-574 complex
23564	OXC5816	UK	2011	gastroenteritis	human stool	*Campylobacter jejuni*	4	7	10	4	1	7	1	45	ST-45 complex
23565	OXC5817	UK	2011	gastroenteritis	human stool	*Campylobacter jejuni*	9	2	4	62	4	5	6	257	ST-257 complex
23566	OXC5818	UK	2011	gastroenteritis	human stool	*Campylobacter jejuni*	2	1	5	3	2	1	5	19	ST-21 complex
23567	OXC5819	UK	2011	gastroenteritis	human stool	*Campylobacter jejuni*	2	1	1	3	2	1	5	21	ST-21 complex
23568	OXC5820	UK	2011	gastroenteritis	human stool	*Campylobacter jejuni*	9	2	4	62	4	133	6	990	ST-257 complex
23569	OXC5821	UK	2011	gastroenteritis	human stool	*Campylobacter jejuni*	8	1	6	3	2	1	12	2135	ST-21 complex
23570	OXC5822	UK	2011	gastroenteritis	human stool	*Campylobacter jejuni*	2	1	1	3	2	1	5	21	ST-21 complex
23571	OXC5823	UK	2011	gastroenteritis	human stool	*Campylobacter jejuni*	2	1	21	3	2	1	5	53	ST-21 complex
23572	OXC5824	UK	2011	gastroenteritis	human stool	*Campylobacter jejuni*	7	71	5	321	11	67	6	3268	
23573	OXC5826	UK	2011	gastroenteritis	human stool	*Campylobacter jejuni*	2	1	1	3	2	1	5	21	ST-21 complex
23574	OXC5827	UK	2011	gastroenteritis	human stool	*Campylobacter coli*	33	39	30	79	113	47	17	860	ST-828 complex
23575	OXC5829	UK	2011	gastroenteritis	human stool	*Campylobacter jejuni*	2	1	12	3	2	1	5	50	ST-21 complex
23576	OXC5830	UK	2011	gastroenteritis	human stool	*Campylobacter jejuni*	8	17	5	2	10	59	6	400	ST-353 complex
23577	OXC5831	UK	2011	gastroenteritis	human stool	*Campylobacter coli*	33	39	30	82	189	47	17	1191	ST-828 complex
23578	OXC5834	UK	2011	gastroenteritis	human stool	*Campylobacter jejuni*	24	2	2	2	10	3	3	5136	ST-464 complex
23579	OXC5835	UK	2011	gastroenteritis	human stool	*Campylobacter jejuni*	2	1	1	3	2	1	5	21	ST-21 complex
23580	OXC5837	UK	2011	gastroenteritis	human stool	*Campylobacter coli*	33	39	30	82	104	56	17	827	ST-828 complex
23581	OXC5838	UK	2011	gastroenteritis	human stool	*Campylobacter jejuni*	2	1	5	2	7	1	5	5173	ST-48 complex
23582	OXC5839	UK	2011	gastroenteritis	human stool	*Campylobacter jejuni*	7	17	5	2	13	3	6	3510	ST-353 complex
23583	OXC5840	UK	2011	gastroenteritis	human stool	*Campylobacter jejuni*	2	1	5	3	2	1	5	19	ST-21 complex
23584	OXC5841	UK	2011	gastroenteritis	human stool	*Campylobacter jejuni*	8	1	6	3	2	1	12	2135	ST-21 complex
23585	OXC5842	UK	2011	gastroenteritis	human stool	*Campylobacter coli*	33	39	66	82	104	44	174	2178	ST-828 complex
23586	OXC5843	UK	2011	gastroenteritis	human stool	*Campylobacter jejuni*	2	1	42	3	148	1	5	861	ST-21 complex
23587	OXC5844	UK	2011	gastroenteritis	human stool	*Campylobacter jejuni*	1	4	2	2	6	3	17	61	ST-61 complex
23588	OXC5846	UK	2011	gastroenteritis	human stool	*Campylobacter jejuni*	2	1	1	3	2	1	5	21	ST-21 complex
23589	OXC5847	UK	2011	gastroenteritis	human stool	*Campylobacter jejuni*	8	10	2	210	11	12	6	2033	ST-354 complex
23590	OXC5848	UK	2011	gastroenteritis	human stool	*Campylobacter jejuni*	2	1	1	3	2	1	5	21	ST-21 complex
23591	OXC5849	UK	2011	gastroenteritis	human stool	*Campylobacter coli*	33	39	30	79	104	47	17	830	ST-828 complex
23592	OXC5851	UK	2011	gastroenteritis	human stool	*Campylobacter jejuni*	24	2	2	2	10	3	1	464	ST-464 complex
23593	OXC5853	UK	2011	gastroenteritis	human stool	*Campylobacter coli*	33	39	30	79	104	47	17	830	ST-828 complex
23594	OXC5854	UK	2011	gastroenteritis	human stool	*Campylobacter jejuni*	3	1	5	17	11	11	6	49	ST-49 complex
23595	OXC5855	UK	2011	gastroenteritis	human stool	*Campylobacter coli*	33	39	30	82	113	47	17	825	ST-828 complex

23596	OXC5856	UK	2011	gastroenteritis	human stool	*Campylobacter coli*	33	39	30	79	104	47	17	830	ST-828 complex
23597	OXC5857	UK	2011	gastroenteritis	human stool	*Campylobacter jejuni*	9	25	2	10	23	3	6	775	ST-52 complex
23598	OXC5858	UK	2011	gastroenteritis	human stool	*Campylobacter jejuni*	7	71	2	62	11	34	6	5244	
23599	OXC5859	UK	2011	gastroenteritis	human stool	*Campylobacter jejuni*	9	2	4	62	4	5	12	2030	ST-257 complex
23600	OXC5860	UK	2011	gastroenteritis	human stool	*Campylobacter jejuni*	1	4	2	2	6	3	17	61	ST-61 complex
23601	OXC5863	UK	2011	gastroenteritis	human stool	*Campylobacter jejuni*	24	2	2	2	10	3	1	464	ST-464 complex
23602	OXC5864	UK	2011	gastroenteritis	human stool	*Campylobacter coli*	33	39	30	82	189	47	17	1191	ST-828 complex
23603	OXC5865	UK	2011	gastroenteritis	human stool	*Campylobacter jejuni*	9	2	4	62	4	5	12	2030	ST-257 complex
23604	OXC5866	UK	2011	gastroenteritis	human stool	*Campylobacter jejuni*	9	2	4	62	4	5	6	257	ST-257 complex
23605	OXC5869	UK	2011	gastroenteritis	human stool	*Campylobacter jejuni*	9	2	4	62	4	5	12	2030	ST-257 complex
23606	OXC5870	UK	2011	gastroenteritis	human stool	*Campylobacter coli*	33	39	30	139	113	47	17	4425	ST-828 complex
23607	OXC5872	UK	2011	gastroenteritis	human stool	*Campylobacter jejuni*	9	2	4	62	4	5	6	257	ST-257 complex
23608	OXC5873	UK	2011	gastroenteritis	human stool	*Campylobacter jejuni*	7	17	2	15	23	3	12	51	ST-443 complex
23609	OXC5874	UK	2011	gastroenteritis	human stool	*Campylobacter jejuni*	2	4	1	2	7	1	5	48	ST-48 complex
23610	OXC5877	UK	2011	gastroenteritis	human stool	*Campylobacter jejuni*	2	4	1	4	19	62	5	475	ST-48 complex
23611	OXC5878	UK	2011	gastroenteritis	human stool	*Campylobacter jejuni*	2	4	1	2	7	1	5	48	ST-48 complex
23612	OXC5879	UK	2011	gastroenteritis	human stool	*Campylobacter jejuni*	2	1	12	3	2	1	5	50	ST-21 complex
23613	OXC5880	UK	2011	gastroenteritis	human stool	*Campylobacter jejuni*	2	1	21	3	2	1	5	53	ST-21 complex
23614	OXC5882	UK	2011	gastroenteritis	human stool	*Campylobacter jejuni*	14	45	2	4	19	3	6	312	ST-658 complex
23615	OXC5884	UK	2011	gastroenteritis	human stool	*Campylobacter jejuni*	2	427	5	37	60	1	5	5819	ST-206 complex
23616	OXC5885	UK	2011	gastroenteritis	human stool	*Campylobacter jejuni*	9	2	4	62	4	5	6	257	ST-257 complex
23617	OXC5886	UK	2011	gastroenteritis	human stool	*Campylobacter jejuni*	7	2	5	2	10	3	6	5	ST-353 complex
23618	OXC5887	UK	2011	gastroenteritis	human stool	*Campylobacter jejuni*	2	1	5	3	2	1	5	19	ST-21 complex
23619	OXC5888	UK	2011	gastroenteritis	human stool	*Campylobacter jejuni*	8	1	6	3	2	1	12	2135	ST-21 complex
23620	OXC5889	UK	2011	gastroenteritis	human stool	*Campylobacter jejuni*	9	2	4	62	4	5	12	2030	ST-257 complex
23621	OXC5890	UK	2011	gastroenteritis	human stool	*Campylobacter jejuni*	1	2	3	4	5	9	3	42	ST-42 complex
23622	OXC5891	UK	2011	gastroenteritis	human stool	*Campylobacter jejuni*	62	4	5	2	2	1	5	572	ST-206 complex
23623	OXC5892	UK	2011	gastroenteritis	human stool	*Campylobacter jejuni*	2	4	5	25	11	3	5	2304	
23624	OXC5894	UK	2011	gastroenteritis	human stool	*Campylobacter jejuni*	7	28	4	28	17	34	12	573	ST-573 complex
23625	OXC5895	UK	2011	gastroenteritis	human stool	*Campylobacter jejuni*	8	1	6	3	2	1	12	2135	ST-21 complex
23626	OXC5896	UK	2011	gastroenteritis	human stool	*Campylobacter sp.*									
23627	OXC5897	UK	2011	gastroenteritis	human stool	*Campylobacter jejuni*	7	2	5	72	2	383	6	4684	
23628	OXC5898	UK	2011	gastroenteritis	human stool	*Campylobacter jejuni*	9	25	2	10	22	3	6	52	ST-52 complex

23629	OXC5900	UK	2011	gastroenteritis	human stool	*Campylobacter jejuni*	9	25	2	10	22	3	6	52	ST-52 complex
23630	OXC5903	UK	2011	gastroenteritis	human stool	*Campylobacter jejuni*	9	2	4	62	4	5	6	257	ST-257 complex
23631	OXC5905	UK	2011	gastroenteritis	human stool	*Campylobacter jejuni*	2	1	12	3	2	1	5	50	ST-21 complex
23632	OXC5906	UK	2011	gastroenteritis	human stool	*Campylobacter jejuni*	2	1	12	3	2	1	5	50	ST-21 complex
23633	OXC5907	UK	2011	gastroenteritis	human stool	*Campylobacter jejuni*	9	2	4	62	4	5	6	257	ST-257 complex
23634	OXC5908	UK	2011	gastroenteritis	human stool	*Campylobacter jejuni*	4	7	40	4	42	51	1	267	ST-283 complex
23635	OXC5909	UK	2011	gastroenteritis	human stool	*Campylobacter jejuni*	2	1	21	3	2	1	5	53	ST-21 complex
23636	OXC5910	UK	2011	gastroenteritis	human stool	*Campylobacter jejuni*	24	2	2	2	10	3	1	464	ST-464 complex
23637	OXC5911	UK	2011	gastroenteritis	human stool	*Campylobacter jejuni*	7	17	27	10	11	3	6	5247	ST-353 complex
23638	OXC5912	UK	2011	gastroenteritis	human stool	*Campylobacter jejuni*	10	27	16	19	10	5	7	403	ST-403 complex
23639	OXC5914	UK	2011	gastroenteritis	human stool	*Campylobacter jejuni*	7	53	2	10	11	3	3	574	ST-574 complex
23640	OXC5915	UK	2011	gastroenteritis	human stool	*Campylobacter jejuni*	9	2	4	62	4	5	6	257	ST-257 complex
23641	OXC5916	UK	2011	gastroenteritis	human stool	*Campylobacter jejuni*	9	2	4	62	4	133	6	990	ST-257 complex
23642	OXC5917	UK	2011	gastroenteritis	human stool	*Campylobacter jejuni*	9	25	2	10	22	3	6	52	ST-52 complex
23643	OXC5918	UK	2011	gastroenteritis	human stool	*Campylobacter jejuni*	2	1	21	3	2	1	5	53	ST-21 complex
23644	OXC5921	UK	2011	gastroenteritis	human stool	*Campylobacter jejuni*	24	2	2	2	10	3	3	5136	ST-464 complex
23645	OXC5922	UK	2011	gastroenteritis	human stool	*Campylobacter coli*	33	39	30	82	104	56	17	827	ST-828 complex
23646	OXC5923	UK	2011	gastroenteritis	human stool	*Campylobacter coli*	33	39	30	82	104	56	17	827	ST-828 complex
23647	OXC5925	UK	2011	gastroenteritis	human stool	*Campylobacter jejuni*	2	1	12	3	2	1	5	50	ST-21 complex
23648	OXC5926	UK	2011	gastroenteritis	human stool	*Campylobacter jejuni*	2	1	12	3	2	1	5	50	ST-21 complex
23649	OXC5927	UK	2011	gastroenteritis	human stool	*Campylobacter jejuni*	8	10	2	210	11	12	6	2033	ST-354 complex
23650	OXC5928	UK	2011	gastroenteritis	human stool	*Campylobacter jejuni*	7	17	2	15	23	3	1	6602	ST-443 complex
23651	OXC5930	UK	2011	gastroenteritis	human stool	*Campylobacter jejuni*	2	4	1	2	7	1	5	48	ST-48 complex
23652	OXC5931	UK	2011	gastroenteritis	human stool	*Campylobacter jejuni*	7	17	5	2	10	3	6	353	ST-353 complex
23653	OXC5932	UK	2011	gastroenteritis	human stool	*Campylobacter jejuni*	9	2	4	62	4	5	12	2030	ST-257 complex
23654	OXC5933	UK	2011	gastroenteritis	human stool	*Campylobacter jejuni*	1	3	6	4	3	3	3	22	ST-22 complex
23655	OXC5934	UK	2011	gastroenteritis	human stool	*Campylobacter jejuni*	7	227	5	72	10	3	1	2084	ST-353 complex
23656	OXC5938	UK	2011	gastroenteritis	human stool	*Campylobacter jejuni*	2	1	1	3	2	1	5	21	ST-21 complex
23657	OXC5939	UK	2011	gastroenteritis	human stool	*Campylobacter jejuni*	2	21	5	37	2	1	5	206	ST-206 complex
23658	OXC5483	UK	2010	gastroenteritis	human stool	*Campylobacter jejuni*	2	4	1	2	7	1	5	48	ST-48 complex
23659	OXC5794	UK	2011	gastroenteritis	human stool	*Campylobacter jejuni*	9	2	4	62	4	5	12	2030	ST-257 complex
23660	OXC5804	UK	2011	gastroenteritis	human stool	*Campylobacter jejuni*	2	1	21	3	2	1	5	53	ST-21 complex
23662	OXC5825	UK	2011	gastroenteritis	human stool	*Campylobacter jejuni*	2	1	21	3	2	1	5	53	ST-21 complex

23663	OXC5828	UK	2011	gastroenteritis	human stool	*Campylobacter jejuni*		2	4	62	4	5	12	
23664	OXC5832	UK	2011	gastroenteritis	human stool	*Campylobacter jejuni*	2	1	1	3	2	1	5	21	ST-21 complex
23665	OXC5833	UK	2011	gastroenteritis	human stool	*Campylobacter jejuni*	9	2	4	510	4	5	12	6603	ST-257 complex
23666	OXC5845	UK	2011	gastroenteritis	human stool	*Campylobacter jejuni*	24	30	5	2	89	3	6	4403	ST-460 complex
23667	OXC5850	UK	2011	gastroenteritis	human stool	*Campylobacter jejuni*	4	7	10	4	1	7	1	45	ST-45 complex
23668	OXC5852	UK	2011	gastroenteritis	human stool	*Campylobacter jejuni*	2	4	2	4	19	3	6	658	ST-658 complex
23669	OXC5861	UK	2011	gastroenteritis	human stool	*Campylobacter jejuni*	2	4	1	2	7	1	5	48	ST-48 complex
23670	OXC5862	UK	2011	gastroenteritis	human stool	*Campylobacter jejuni*	2	1	1	3	2	1	5	21	ST-21 complex
23671	OXC5871	UK	2011	gastroenteritis	human stool	*Campylobacter jejuni*	2	1	5	3	2	1	5	19	ST-21 complex
23672	OXC5875	UK	2011	gastroenteritis	human stool	*Campylobacter jejuni*	1	4	2	2	6	3	17	61	ST-61 complex
23673	OXC5876	UK	2011	gastroenteritis	human stool	*Campylobacter jejuni*	2	4	1	437	11	3	6	5245	ST-658 complex
23674	OXC5883	UK	2011	gastroenteritis	human stool	*Campylobacter jejuni*	9	2	2	2	568	5	6	6604	ST-257 complex
23675	OXC5893	UK	2011	gastroenteritis	human stool	*Campylobacter jejuni*	10	27	16	19	10	5	130	5246	ST-403 complex
23676	OXC5899	UK	2011	gastroenteritis	human stool	*Campylobacter jejuni*	2	1	5	3	2	1	5	19	ST-21 complex
23677	OXC5902	UK	2011	gastroenteritis	human stool	*Campylobacter jejuni*	2	1	12	3	2	1	5	50	ST-21 complex
23678	OXC5904	UK	2011	gastroenteritis	human stool	*Campylobacter jejuni*	91	2	42	4	169	9	8	986	
23679	OXC5924	UK	2011	gastroenteritis	human stool	*Campylobacter jejuni*	2	1	1	3	2	1	5	21	ST-21 complex
23680	OXC5929	UK	2011	gastroenteritis	human stool	*Campylobacter jejuni*	2	1	1	3	2	1	5	21	ST-21 complex
23681	OXC5937	UK	2011	gastroenteritis	human stool	*Campylobacter jejuni*	2	1	1	3	2	1	5	21	ST-21 complex
23682	OXC5940	UK	2011	gastroenteritis	human stool	*Campylobacter jejuni*	24	2	2	2	10	3	1	464	ST-464 complex
23683	OXC5941	UK	2011	gastroenteritis	human stool	*Campylobacter jejuni*	4	7	10	4	1	7	1	45	ST-45 complex

*Isolate genomes and metadata downloaded from pubMLST.org/Campylobacter

Table S3: List of biogeographical epidemiological markers, including lists of highly recombining genes as determined by pairwise analysis of nucleotide diversity (more than 2% diversity); and genes used to model biogeographical segregation in structure.

Locus ID	Gene name	Description	Comment	Used in attribution model
Cj0024	*Cj0024*	nrdA, ribonucleoside-diphosphate reductase alpha chain		
Cj0031	*Cj0031*	putative type IIS restriction/modification enzyme	phase variable	
Cj0034c	*Cj0034c*	putative periplasmic protein		✓
Cj0035c	*Cj0035c*	putative efflux protein	fluoroquinoline resistance	
Cj0036	*Cj0036*	hypothetical protein		
Cj0038c	*Cj0038c*	putative poly(A) polymerase family protein		
Cj0138	*Cj0138*	conserved hypothetical protein		✓
Cj0141c	*Cj0141c*	putative ABC transporter integral membrane protein	manganese transport system	
Cj0537	*oorB*	OORB subunit of 2-oxoglutarate:acceptor oxidoreductase		
Cj0538	*fliD*	flagellar hook-associated protein		
Cj0613	*pstS*	putative periplasmic phosphate binding protein		✓
Cj0614	*pstC*	putative phosphate transport system permease protein		✓
Cj0615	*pstA*	putative phosphate transport system permease protein		
Cj0616	*pstB*	phosphate transport ATP-binding protein		
Cj0619	*Cj0619*	putative MATE family transport protein: fluoroquinolone resist	fluoroquinoline resistance	
Cj0621	*Cj0621*	hypothetical protein		
Cj0622	*hypF*	carbamoyltransferase		
Cj0623	*hypB*	hydrogenase isoenzymes formation protein		
Cj0624	*hypC*	hydrogenase isoenzymes formation protein		
Cj0625	*hypD*	hydrogenase isoenzymes formation protein		
Cj0626	*hypE*	hydrogenase isoenzymes formation protein		
Cj0627	*hypA*	hydrogenase expression/formation protein		
Cj0630c	*Cj0630c*	putative DNA polymerase III		
Cj0631c	*Cj0631c*	putative ribonuclease		
Cj0632	*ilvC*	ketol-acid reductoisomerase		
Cj0633	*Cj0633*	putative periplasmic protein		
Cj0634	*dprA*	DNA processing protein A		
Cj0635	*Cj0635*	putative Holliday junction resolvase		✓
Cj0636	*Cj0636*	NOL1/NOP2/sun family protein		
Cj1050c	*npdA*	NAD-dependent deacetylase		✓
Cj1052c	*mutS*	putative mismatch repair protein		
Cj1053c	*Cj1053c*	putative integral membrane protein		
Cj1054c	*murC*	UDP-N-acetylmuramate--alanine ligase		
Cj1056c	*Cj1056c*	putative carbon-nitrogen hydrolase family protein		
Cj1058c	*guaB*	inosine-5'-monophosphate dehydrogenase		
Cj1066	*rdxA*	nitroreductase		
Cj1067	*pgsA*	CDP-diacylglycerol--glycerol-3-phosphate 3-phosphatidyltransferase		
Cj1068	*Cj1068*	putative peptidase M50 family protein		
Cj1069	*Cj1069*	conserved hypothetical protein		
Cj1151c	*hldD*	ADP-glyceromanno-heptose 6-epimerase	LOS	
Cj1157c	*dnaX*	putative DNA polymerase III subunit gamma		
Cj1161c	*Cj1161c*	putative cation-transporting ATPase		
Cj1163c	*Cj1163c*	putative cation transport protein	cobalt-zinc-cadmium resistance protein CZCD	
Cj1166c	*Cj1166c*	putative integral membrane protein		
Cj1171c	*ppi*	peptidyl-prolyl cis-trans isomerase		
Cj1172c	*Cj1172c*	conserved hypothetical protein		
Cj1174	*Cj1174*	putative efflux protein (multidrug resistance protein)	fluoroquinoline resistance	
Cj1175c	*argS*	arginyl-tRNA synthetase		
Cj1259	*porA*	major outer membrane protein	MLST	
Cj1313	*pseH*	N-acetyltransferase specific for PseC product, UDP-4-amino-4,6-dideoxy-beta-L-AltNAc	O-linked glycosylation locus (Cj1293 - Cj1342)	✓
Cj1343c	*Cj1343c*	putative periplasmic protein		
Cj1344c	*Cj1344c*	putative glycoprotease	O-sialoglycoprotein endopeptidase	
Cj1345c	*Cj1345c*	putative periplasmic protein	glycoprotein	
Cj1398	*feoB*	ferrous iron transport protein		
Cj1407c	*Cj1407c*	putative phospho-sugar mutase		
Cj1411c	*Cj1411c*	putative cytochrome P450		
Cj1561	*Cj1561*	putative transcriptional regulator	arsenical resistance operon repressor	
